# Pervasive context-dependent effects in the genetic architecture of complex and quantitative traits revealed by a powerful multiparent mapping population in yeast

**DOI:** 10.1371/journal.pgen.1012288

**Published:** 2026-08-27

**Authors:** Gareth A. Cromie, Russell S. Lo, Lauren Ames, Trey S. Morgan, Katherine Owens, Anne E. Clark, Martin S. Timour, Julee Ashmead, Michelle Tang, J. Nathan Kutz, Joshua M. Akey, Aimée M. Dudley

**Affiliations:** 1 Pacific Northwest Research Institute, Seattle, Washington, United States of America; 2 Department of Applied Mathematics, University of Washington, Seattle, Washington, United States of America; 3 Department of Genome Sciences, University of Washington, Seattle, Washington, United States of America; 4 Lewis Sigler Institute, Princeton University, Princeton, New Jersey, United States of America; USC, UNITED STATES OF AMERICA

## Abstract

The genetic dissection of complex traits remains a major challenge in basic and biomedical research, but is essential for understanding the molecular pathways that shape phenotypic variation and for developing predictive models of trait and disease susceptibility. Here, we leverage a novel multiparent mapping population of budding yeast, CYClones, comprising 9,344 haploid strains derived from eight genetically diverse founders (~270,000 SNVs, ~ 1 per 44 bp, capturing 56% of common variants and 32% of all variants with a minor allele frequency greater than 0.005 in the global population), to identify quantitative trait loci (QTL) and systematically investigate the genetic architecture of growth rates across ten environmental conditions. In total, we identified 349 QTL (ranging from 18 to 49 QTL per growth condition) that explained between 60% and 100% of narrow sense heritability across traits. The high power and resolution of CYClones revealed that growth traits exhibited distinct, condition-specific genetic architectures with extensive allelic heterogeneity, where a QTL was the result of multiple tightly linked causal variants. We also observed pleiotropy among QTL with complex, trait-dependent allele effects that are also consistent with allelic heterogeneity. Genetic complexity varied widely, with some traits showing nearly Mendelian architectures, while others were highly polygenic. Introgressed loci played a prominent role in the landscape of growth rate QTL, including a QTL localized to a 2.4 kb interval in the *PCA1* cadmium transporter that explains 72% of variation in cadmium resistance and is largely driven by an introgression, and a non-additive interaction between the *GAL3* regulator and introgressed *GAL1/7/10* alleles, extending a previously described three-locus GAL-pathway incompatibility to a four-locus interaction. In both cadmium and galactose conditions, we show that allelic variation at a small number of loci stratifies the population into regulatory or physiological subgroups, each with distinct genetic architectures, a specific manifestation of epistasis we term allele-dependent stratification. Collectively, our results provide novel insights into the genetics of growth rates in budding yeast, the architectural features of genetic complexity, and demonstrate that CYClones is a powerful platform for revealing the molecular basis of complex trait variation.

## Author summary

Most traits that matter, such as how tall a plant grows, how a microbe resists a drug, or whether a person develops a disease, are shaped by many genes acting together, each contributing a small effect. Identifying those genes, and understanding how they combine, remains a central challenge in genetics. To address this, we built an unusually powerful genetic resource in baker’s yeast: a population of more than 9,000 genetically distinct strains bred from eight diverse natural founders. Because we determined the complete genome of every strain, after measuring how well each one grew under ten different conditions (including several drugs and stressors) we could trace the genetic differences underlying these traits back to specific genomic regions. We identified 349 trait-linked regions and, thanks to the large sample size, narrowed many of them to individual genes. Some traits were dominated by variation in single genes, while others reflected smaller contributions from many genes. Often the identified regions held several distinct genetic changes affecting the trait, rather than one. Two examples, natural differences in using the sugar galactose and in resisting toxic cadmium, show how a few key variants can sort the strains into subgroups, each shaped by different genes.

## Introduction

Delineating the genetic architecture of complex traits is a fundamentally important problem with broad significance in genetics, evolution, and personalized medicine [1]. A deeper understanding of the relationship between genotypes and phenotypes will help elucidate the biological mechanisms contributing to variation in complex traits and enable phenotypes to be accurately predicted from an individual’s genome sequence. Mendelian traits and diseases are already routinely predicted from DNA sequences, such as in newborn screening programs [2]. Extending this paradigm to the prediction of complex traits and disease susceptibility, through polygenic risk scoring, has the potential to revolutionize medicine. However, polygenic scores have a number of limitations that need to be overcome, including accuracy and portability across populations, before being broadly useful [3].

Although large biobank-scale cohorts, such as the UK Biobank [4] and All of Us Project [5], have facilitated powerful genome-wide association studies (GWAS) in humans, understanding the rules governing genotype-phenotype relationships in outbred populations, like humans, is challenging. Specifically, high levels of allelic diversity and linkage disequilibrium in humans make identifying causal alleles difficult, particularly rare variants with small effect sizes [6]. Additionally, context-dependent effects, including gene x gene and gene x environment interactions, have proven difficult to assess in humans. Thus, model organisms have been a useful complementary strategy to understand the genetic architecture of complex traits. Large mapping populations derived from crossing multiple divergent parents have emerged as a popular study design to investigate the fundamental characteristics of genotype-phenotype maps [7–10]. These multiparent study designs, modeled after the Mouse Collaborative Cross [11], are powerful because they capture a larger proportion of population diversity compared to simpler crosses of two parental strains, and thus have a genetic background that more closely recapitulates outbred populations, while avoiding complications from rare alleles and the confounding effects of population structure [12].

Budding yeast (*Saccharomyces cerevisiae*), with its rapid growth, compact and well-studied genome, and an unusually high meiotic recombination rate of approximately 45 crossovers per genome per meiosis [13], is a particularly attractive model organism for genetic mapping. The combination of these features enables the construction of mapping populations that are very large [14–16], deeply recombined, and tractable for propagation and phenotyping. Yeast linkage mapping was pioneered with biparental crosses such as BY × RM [17,18], which demonstrated that increasing the number of segregants improved the power to detect small-effect QTL. Subsequent advances, including X-QTL approaches that use selective pressure on millions of pooled segregants [19] and the recent barcoded bulk QTL approach that genotypes ~100,000 individuals from a single cross [20], have further extended the scale and resolution achievable in biparental designs. However, biparental crosses sample only two haplotypes per locus, limiting the diversity of alleles and genetic backgrounds that can be examined in a single experiment.

To extend allelic diversity beyond two founders, several yeast multiparent populations have been constructed. The four-parent SGRP-4X advanced intercross provided proof-of-principle at modest scale [21]. A 12-founder round-robin mapped complex traits across diverse *S. cerevisiae* isolates [22]. A 16-founder cross of approximately 14,000 segregants demonstrated that rare variants in the founder pool can contribute disproportionately to phenotypic variance [14], and two synthetic 18-way outcrossed populations carried through 12 generations of random mating maintained haplotype contributions from at least 10 of the 18 founders across more than 99% of the genome [23]. A related advanced-intercross resource, the Mitonuclear Recombinant Collection, was constructed specifically to map the nuclear and mitochondrial loci underlying mitonuclear interactions [24]. Diallel panels of inter-isolate hybrids offer a complementary strategy, mapping low-frequency variants through pairwise hybridisation, with recent designs incorporating long-read sequencing to capture structural variants alongside SNPs [25,26]. Together, these resources have established yeast as a leading system for the dissection of complex traits, but each design imposes a distinct trade-off between scale, allelic diversity, individual- versus pooled-strain phenotyping, and the variant types resolved.

To this end, we constructed the CYClones (Collaborative Yeast Cross clones) mapping population using a multiparent funnel cross design analogous to the Mouse Collaborative Cross. The final population comprises 9,344 fully haplotyped strains, each individually low-coverage sequenced and successfully phenotyped, with construction details and statistical-power assessment described in a companion paper (https://doi.org/10.1101/2025.10.15.682626). We measured quantitative growth rates for all CYClones strains across ten media conditions chosen to span evolutionarily, ecologically, and clinically relevant environments, and used these data to estimate heritability, map quantitative trait loci (QTL), and characterize the genetic architecture of each trait. Our results reveal pervasive context-dependent effects, widespread allelic heterogeneity at QTL, and a specific manifestation of epistasis we term *allele-dependent stratification*, in which a small number of large-effect alleles partition the population into subgroups with qualitatively distinct genetic architectures. Together, these findings establish CYClones as a powerful platform for the mechanistic dissection of complex-trait variation in yeast and reveal architectural features likely to be general.

## Materials and methods

### Media and genetic manipulation of yeast

Unless noted, standard media and methods were used for the growth and manipulation of yeast. Unless specified, yeast strains were grown in YPD medium (1% yeast extract, 2% bacto peptone, 2% glucose), with 2% agar for solid plates. Nourseothricin (NAT) was used at a final concentration of 100ug/ml. Hygromycin (HYG) was used at a final concentration of 500ug/ml.

### Condition specific growth of the mapping population

Yeast growth assays were carried out on solid media on Nunc OmniTrays. With the exception of the galactose condition, all media were YPD (2% glucose) agar supplemented with the appropriate stock solutions (Table #1 in https://doi.org/10.5281/zenodo.17381713). Galactose medium was YPGal solid medium containing the respiratory inhibitor antimycin A (1% yeast extract, 2% bacto peptone, 2% galactose, 1µg/ml antimycinA). Plates contained 35 ml of agar media dispensed by an automated plate pourer (Kreo Technologies). Plates were labeled with a computer-readable barcode to facilitate automated plate identification and dried at room temperature for three days before use. Each media condition was assayed twice, with biological replicates being performed on different days and with independently generated source plates.

Strains were robotically pinned in duplicate from source plates onto the assay plates, photographed at 24, 48 and 72 h, and colony area was extracted from the images using PyPlate, utilizing intensity thresholding [27] and circle detection [28]  (full pinning and image-analysis procedures in S1 Text). Final phenotypes were obtained by normalizing the colony-area measurements for multiplicative plate, weekly-batch and plate-edge effects and averaging across replicates (S1 Text).

### Broad and narrow sense heritability

Broad sense heritability was calculated as *H*^*2*^ = 1−(σ^2^_e_/σ^2^_s_), where σ^2^_s_ is the variance of the population of segregant means, and σ^2^_e_ is the variance of the sampling distribution of these means. We calculated σ^2^_e_ based on the variance of duplicate measures of the same strain, using a random effects ANOVA implemented using the ‘lmer’ function in the R package “lme4” [29].

Narrow sense heritability was calculated using the R package “rrBLUP” [30]. First, a covariance matrix, K, was calculated using the ‘A.mat’ function applied to a matrix of allele values for all strains at the set of scaffolding markers. To produce the allele matrix, haplotypes were converted back into binary allele calls, with major alleles encoded with the value “1”, and minor alleles with the value “0”. Next, the ‘mixed.solve’ function was used with the K matrix and the vector of observed phenotypes for each condition at each timepoint to calculate additive (V_a_) and residual (V_e_) variance components. The narrow sense heritability for each condition at each timepoint was then estimated as V_a_/(V_a_ + V_e_). Standard errors were then calculated as the standard deviation of 100 bootstrap replicates of each time + condition.

### QTL mapping

For single-marker linkage mapping, at each tested position, the LOD score was calculated as LOD = n/2 x log_10_(RSS_0_/RSS_1_), where RSS_0_ is the null residual sum of squares assuming no QTL (single population mean) and RSS_1_ is the residual sum of squares assuming a QTL (fitting a separate mean for the 8 subpopulations defined by the haplotype at the current marker).

For pairwise-marker linkage mapping of genetic interactions, at each tested pair of positions (*s*,*t*) the LOD_*i*_ score, measuring the improvement in the fit of a model allowing interaction (L_*f*_) over an additive model (L_*a*_), was calculated as L_*f*_(*s*,*t*) – L_*a*_(*s*,*t*), where L_*f*_(*s*,*t*) and L_*a*_(*s*,*t*) are the log_10_ likelihoods for the interaction and additive models, respectively. Likelihoods were calculated from the residuals of each model, assuming normality.

We used permutations to determine a LOD score threshold that corrected for both the number of markers tested in each genome-wide scan and the total number of phenotypes analyzed. Specifically, we carried out 1000 permutations of which 10-phenotype vector (from the 72 h dataset) was associated with each haplotype vector. We then carried out single marker linkage analysis as above and identified the highest LOD score across all phenotypes for that permutation. The 95^th^ percentile of the 1000 permutation maximum LOD values was used to define a genome-wide, phenotype-corrected 5% FWER LOD score of 8.5.

Because estimating family-wise error rates for the pairwise marker analyses was computationally intensive, instead a 0.1% false discovery rate (FDR) was calculated for each of the pairwise non-additive datasets using a null distribution for each condition at each timepoint. The null distribution was generated by calculating the interaction LOD, as above, but using a single permutation of the residuals from the additive model as the phenotype for each pair of markers.

### Generation of additive genetic models and fine mapping of QTL

For each condition and timepoint, additive genetic models were constructed by an iterative process using forward selection. In the first iteration, single-marker LOD scores were calculated for each of the 5479 scaffolding markers, and we retained the most significant marker per chromosome if it exceeded a LOD of 9, conservatively based on the 5% FWER LOD score threshold described above. Then, a linear model was fit using the phenotype values and the haplotypes at the set of identified markers. The residuals from this model were then used as the phenotype values and a second genome-wide linkage analysis was performed, identifying the most significant marker per chromosome, as above. The markers identified from this analysis were then added to the existing set of markers and used to fit a linear model to the phenotype values. We repeated this process until no markers above the LOD score threshold were identified or until an ANOVA, comparing the model from the current iteration to the preceding model, failed to show a significant improvement (p-value>0.001).

The proportion of variance explained by the additive models, and the additional variance captured by allowing pairwise non-additive interactions between the loci of the additive models, was estimated by 10-fold cross-validation (S1 Text).

For the additive models, each QTL was fine-mapped to a 2-LOD confidence interval using the full marker set, and the 24 h and 48 h confidence intervals were used to refine the gene-level resolution of the 72 h QTL (S1 Text).

### Determination of QTL multimodality

Each QTL was tested for polymodality by comparing, under cross-validation, the variance explained by the full eight-haplotype model with that of a binarized two-state recoding (S1 Text).

### Identification of pleiotropic genes

Genes mapped to single-gene resolution in more conditions than expected by chance were identified by permutation (S1 Text).

### GAL3 variant construction and testing

The *GAL3* region of founder strains YO2685 and YO2563 was amplified from genomic DNA and cloned into the centromeric pRS41N plasmid,^19^ via Gibson assembly. Single nucleotide changes in *GAL3* were then induced by Quikchange PCR. All resulting plasmids (Table #4 in https://doi.org/10.5281/zenodo.17381713) were fully sequenced by Oxford Nanopore Sequencing. Plasmids containing *GAL3* variants were transformed into either YO2685 or YO2563 and growth on YPGal medium was quantified, using PyPlate, from images of yeast strains replica pinned onto solid agar plates. Growth was scaled relative to transformation with the S288c reference allele of *GAL3* (100%) and an empty vector (0%) (Fig 4D).

### CAD and GAL Statistical and subpopulation analyses

The *GAL3* pairwise-interaction analysis, the definition of the *GAL3*^+^/*GAL3*^-^ and *GAL1/7/10*-introgression subgroups, and the subgroup and global-versus-subgroup model comparisons are described in S1 Text.

Similarly, the introgression-aware re-haplotyping of the right end of chromosome II, the definition of the *PCA1* subgroups, and their subgroup QTL analyses are also described in S1 Text.

## Results

### Overview of the CYClones mapping population

**Fig 1:**
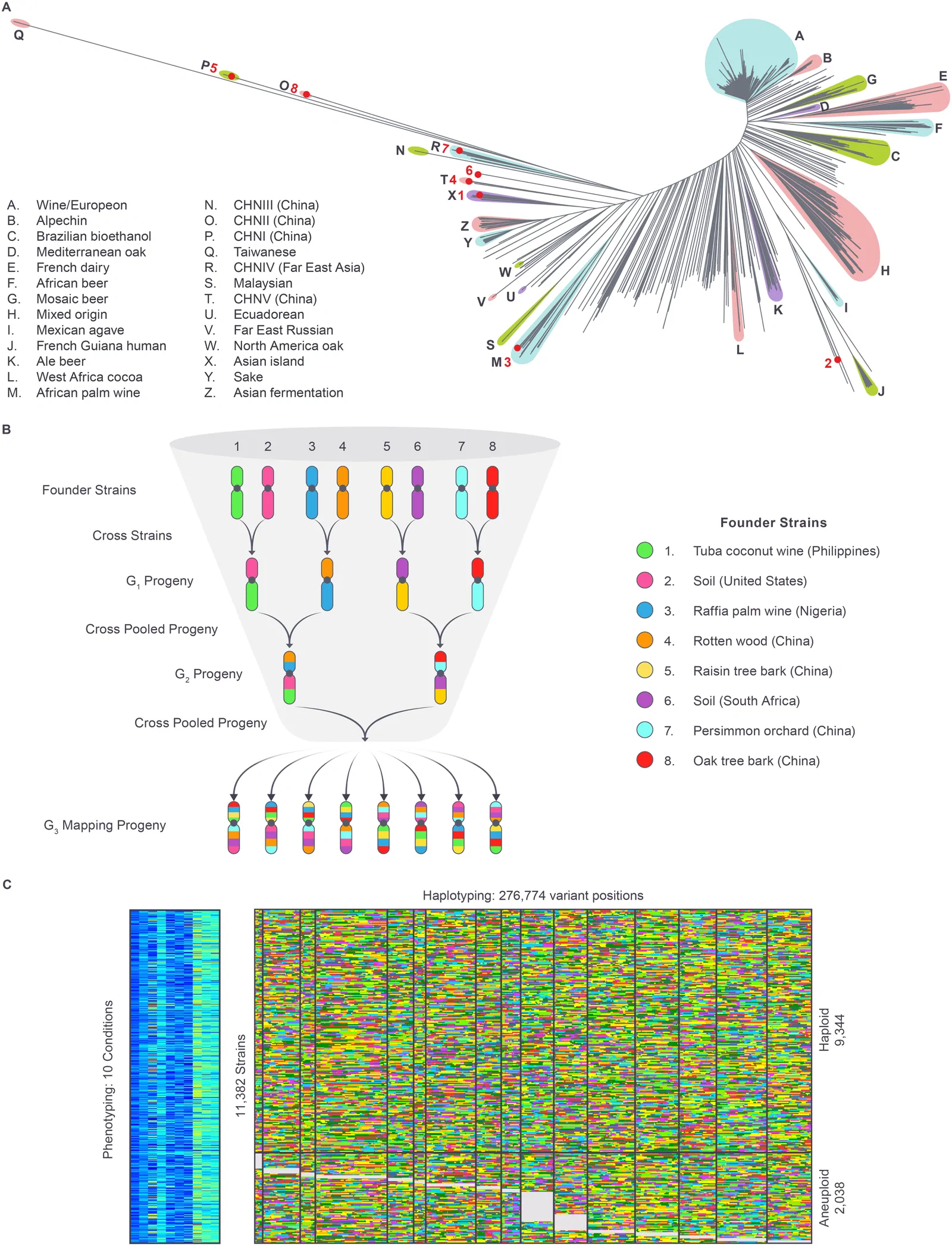
Mapping population and dataset. **(A)** Phylogenetic tree of the global *S. cerevisiae* population with subpopulations from Peter *et al*. [18] and the eight funnel cross founder strains (numbered 1:8 in red) indicated. **(B)** The eight founder strains were used to generate a mapping population via a funnel cross design. Each founder strain was mated pairwise to produce heterozygous diploids, which were each sporulated to produce pools of 576 MAT**a** and 576 MATα haploid progeny. Two rounds of reciprocal crossing using pooled strains were then carried out to produce the final mapping population of 11,484 strains. **(C)** Each of the strains in the final mapping population was individually genotyped and karyotyped by low coverage WGS. Growth of each strain on agar plates was measured on ten different media, in duplicate, and at 3 time points. After quality control filtering, this resulted in a final population of 9,344 euploid and 2,038 aneuploid strains each individually haplotyped, with growth phenotypes measured individually on 10 media conditions. Haplotype colors as in **B**.

To construct the CYClones mapping population, we selected eight haploid “founder” strains isolated from distinct geographic and environmental sources (Fig 1A) and crossed them in a funnel design (Fig 1B). The eight founders were YO2685 (founder 1; tuba palm wine, Philippines), YO2545 (founder 2; soil, Illinois, USA), YO2563 (founder 3; raffia palm wine, Nigeria), YO2552 (founder 4; rotten wood, Hainan, China), YO2633 (founder 5; tree bark, Fujian, China), YO2547 (founder 6; soil, South Africa), YO2553 (founder 7; persimmon orchard, Beijing, China) and YO2588 (founder 8; oak bark, Shaanxi, China), with full strain details in a companion paper (https://doi.org/10.1101/2025.10.15.682626). The eight parental strains were carefully selected to capture a significant proportion of segregating variation in the global budding yeast population (Fig 1A and 1B). Specifically, the eight parental strains are genetically diverse, with ~270k high-quality single nucleotide variants (SNVs) (Table #5 in https://doi.org/10.5281/zenodo.17381713), a density of ~1 SNVs per 44 bases, that include 56% of common variants (minor allele frequencies greater than 0.05) and 32% of all variants, with minor allele frequencies greater than 0.005, in the global *S. cerevisiae* population [18].

Construction of the CYClones library employed three rounds of crossing (Fig 1B) to produce 11,484 haploid strains that are recombinant mosaics of the eight founder haplotypes. Unlike mice or other diploid organisms, these haploid yeast strains did not require further inbreeding to achieve homozygosity. Low coverage whole genome sequence analysis with imputation was used to genotype the ~ 270k founder SNVs in each segregant (Fig 1C and Table #6 in https://doi.org/10.5281/zenodo.17381713) and to identify and exclude aneuploid strains. Of the resulting euploid, fully haplotyped strains, 9,344 were successfully phenotyped and constitute the final mapping population analyzed here. A detailed description of the construction of the CYClones population is provided in a companion publication (https://doi.org/10.1101/2025.10.15.682626). Simulations described in that paper indicate that CYClones is expected to have  ≥ 95% power to identify variants with even very weak phenotypic effects (heritability ≥ 0.36%) at a resolution of a few kb or less, which is often less than the length of a single gene.

In addition to the core marker set of ~270k SNVs used for QTL mapping, the companion study also provides near telomere-to-telomere (T2T) Oxford Nanopore assemblies of all eight founders (File #9; https://doi.org/10.5281/zenodo.17362653). This supplementary resource was not used to generate the core marker set but is provided as a tool for follow-up experiments. Because our haplotype-based linkage mapping localizes QTL to genomic regions rather than to individual candidate variants, it is agnostic to whether the underlying causal change is an SNV, indel, or structural variant. The T2T assemblies therefore serve as a catalogue for prioritizing candidate causative variants of any class at mapped QTL. In addition to SNVs, relative to S288c, these assemblies also identify 866 non-redundant structural variants ≥50 bp (241–320 per founder: 481 insertions, 271 deletions, 68 duplications, 12 translocations, 10 inversions and 24 unclassified).

### High precision phenotyping reveals ten largely independent growth traits

For each of these 9,344 strains, we measured growth rates on ten different media conditions that perturb a diverse set of biological processes representing evolutionary, ecological, and clinically relevant environments (Table #2 in https://doi.org/10.5281/zenodo.17381713). The conditions tested were: rich medium containing the carbon source galactose (GAL; 2% galactose, 1 µg/ml antimycin A), rich medium containing the carbon source glucose (YPD; 2% glucose), YPD supplemented with the antifungal drugs caspofungin (CF, 400 ng/ml) or fluconazole (FLZ, 8 µg/ml), and YPD supplemented with the chemical stressors cadmium chloride (CAD, 100 µM), caffeine (CAF, 10.3 mM), calcium chloride (CAL, 500 mM), hydroxyurea (HU, 145 mM), rapamycin (RAP, 5 ng/ml), or sorbitol (SOR, 1.2 M). Specifically, GAL serves as an evolutionary perturbation: utilization of alternative carbon sources is a classic driver of yeast adaptation, and the GAL regulon has been a major model for evolutionary genetics in budding yeast. SOR (osmotic stress), CAL (ionic and calcium stress), CAD (heavy-metal contamination), and CAF (TOR-pathway inhibitor) probe ecologically relevant stressors that natural yeast strains encounter outside the laboratory. CF and FLZ are clinically relevant antifungal drugs used in the treatment of human yeast infections, and HU and RAP are chemotherapeutic agents (HU in cancer and sickle-cell therapy, RAP as an mTOR inhibitor). YPD provides a baseline rich-medium reference. These conditions therefore sample a broad range of physiological, stress, and ecological perturbations that natural populations of budding yeast might encounter, allowing us to explore the general principles of the genetic architecture of quantitative trait variation. For phenotyping, strains were robotically pinned in duplicate from liquid culture onto solid-media plates. Growth at three timepoints (24, 48 and 72 hours) was then assessed from colony area by automated image analysis (Methods). Reproducibility was very high, with a median *r*^*2*^ value of 0.93 between the independent measurements for each condition at 72 h. The distributions of these growth phenotypes across the mapping population are shown in S9 Fig. As was seen in a previous study measuring the effect on growth of all viable yeast gene deletions under many of the same conditions [31], growth values on the chosen conditions were largely uncorrelated, with a median *r*^*2*^ value of 0.04 (Table #7 in https://doi.org/10.5281/zenodo.17381713), suggesting that distinct biological processes are required for growth under these ten conditions.

### Highly diverse genetic architectures characterize the ten traits

**Fig 2:**
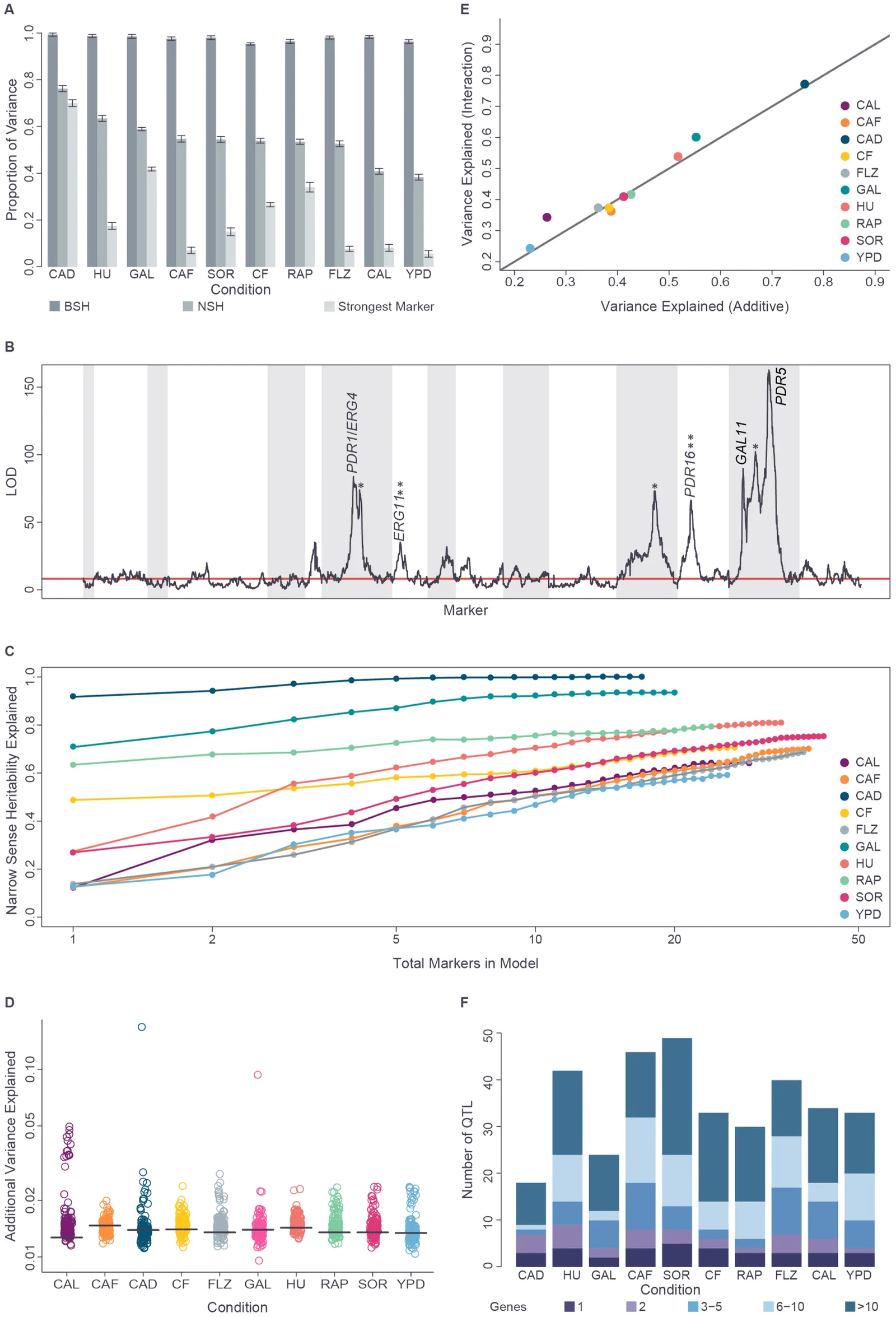
Genetic architecture of ten traits. **(A)** Broad (BSH) and narrow (NSH) heritability, and variance explained by single strongest marker, for all conditions at 72 **h. (B)** Single scaffolding marker LODs for fluconazole, chromosomes alternating grey and white. Known resistance genes mapped to 1, 2 or 3-5 gene (**) resolution are indicated. Single asterisks indicate non-FLZ-specific peaks. **(C)** Proportion narrow sense heritability explained by additive models as QTL are added in order of strength. **(D)** Maximum additional proportion variance beyond additivity explained by inter-chromosomal pairwise interactions with 0.1% FDR thresholds (black horizontal lines). **(E)** Effect on total proportion variance explained of allowing non-additive interactions within additive models. **(F)** Additive model QTL number and resolution (number of genes in 2-LOD interval).

Phenotypic variation was overwhelmingly driven by genetics for all ten conditions, with the estimated proportion of the phenotypic variance explained by genetic effects (broad-sense heritability, H^2^) ranging from 95-99% at 72 h (Fig 2A) and >90% at all timepoints. As such, this dataset should be highly powered to dissect genotype-phenotype associations. We next estimated the proportion of the phenotypic variance explained by purely additive genetic effects (narrow sense heritability, h^2^). The variability of this metric, ranging from 38% (rich medium, YPD) to 76% (cadmium chloride, CAD) at 72 h (Fig 2A), suggested that diverse genetic architectures underlie the ten traits being studied. To characterize these genetic architectures, we performed haplotype-based linkage mapping for each condition at each timepoint using a “scaffolding” subset of 5,479 markers (out of the total set of ~270k markers) spaced approximately 2 kb apart across the genome (Table #3 in https://doi.org/10.5281/zenodo.17381713). Simulations demonstrated that this dense set of scaffolding markers shows very little loss of sensitivity for QTL detection relative to the full set of ~270k markers (described in the companion paper: https://doi.org/10.1101/2025.10.15.682626). The genetic architectures of each condition were consistent across the three timepoints. The rank order of marker LOD scores was extremely concordant between the adjacent 48 h and 72 h timepoints (Spearman ρ = 0.96–0.99) and, as expected, somewhat weaker between the more widely separated 24 h and 72 h timepoints (ρ = 0.67–0.92). We therefore focused subsequent analyses on the 72 h timepoint, when total colony growth was maximal, using the 24 h and 48 h QTL data only to refine QTL resolution at 72 h (Methods).

Further analysis confirmed the power of the 72 h dataset, which was able to detect QTL effects explaining as little as 0.4% of phenotypic variation (5% family-wise significance: LOD = 8.5), a result consistent with simulations which predicted ≥95% power to identify variants with heritability ≥ 0.36% (described in the companion paper: https://doi.org/10.1101/2025.10.15.682626). The ten traits examined were all highly complex, with multiple significant QTL peaks identified in each condition. As expected, substantial variation was observed in the genetic architectures underlying the different traits (S1 Fig and Table #3 in https://doi.org/10.5281/zenodo.17381713). Not only did the positions of the QTL peaks vary between conditions, consistent with the trait-to-trait differences in which genes are sensitive to genetic variation, but the growth phenotypes exhibited markedly distinct degrees of complexity. Some phenotypes, such as growth in GAL (Figs 2A and S1) displayed an almost Mendelian architecture, dominated by a single QTL (max LOD = 1101; 42% of total variance), whereas other conditions, such as FLZ, SOR and YPD were highly polygenic, with the strongest single locus explaining only 7.7%, 15.1% and 5.6% of phenotypic variation, respectively (Figs 2A, 2B and S1). Across the ten traits, the variance explained by the single strongest locus ranged from 5.6% (YPD) to 70.1% (CAD).

### Genetic models built using CYClones can explain most of the additive phenotypic variation across all phenotypes

To explore these architectures further, we developed an additive genetic model for each condition by forward selection using the scaffolding markers (Methods). For all traits, the additive models explained most of the narrow sense heritability (60–100% by 10-fold cross validation). Our results are consistent with Fisher’s infinitesimal model [32], with QTL number increasing as effect size decreases (Fig 2C), similar to the results of a recent large-scale budding yeast barcoded bulk QTL study [20]. However, for several of the conditions (e.g., YPD, FLZ) there is a substantial gap between narrow and broad sense heritability (Fig 2A), indicating that, for these traits, genetically-determined phenotypic variation cannot be fully explained by additive models and suggesting a role for non-additive genetic interactions.

Comparison of additive versus interaction models for all pairs of scaffolding markers in all conditions demonstrated an ability to detect pairwise (non-additive) interactions, down to a (0.1% FDR) threshold of ~1.4% of variance explained (Fig 2D). Significant inter-chromosomal interactions were common but weak: 687 of the 1,200 chromosome-pair maxima across the ten conditions (57%) exceeded the significance threshold, but explained a median of just 1.5% of variance above additivity. Strong interactions were rare, with only the cadmium and galactose conditions having non-additive pairwise interactions exceeding 5% of variance (maximum 16.5%). Extending our additive genetic models to allow non-additive interactions between the markers in those models, showed little improvement in performance, as assessed by 10-fold cross-validation (Fig 2E). This pattern is consistent with results from both human GWAS and model organism studies, where additive models capture most heritable variance, and including pairwise interactions only modestly improves prediction [33–36].

### CYClones enables large numbers of QTL to be mapped at high resolution across diverse genetic architectures

For each trait, we were able to identify large numbers of statistically significant QTL from the additive models, from 18 in CAD to 49 in SOR, totaling 349 QTL across all conditions at 72 h. These QTL were added to the model iteratively using a LOD cutoff of 9 to account for multiple marker and condition testing (Materials and Methods). These QTL were then mapped at high resolution using the full set of ~270k high-quality variant sites, with 60 (17%) mapped to intervals of less than 5 kb and 159 (46%) mapped to intervals of less than 20 kb. Using the mapping data from all three timepoints to further resolve the position of each QTL (Methods), enabled 34 putative causal alleles to be mapped to single gene resolution in the 72 h dataset. An additional 29 and 22 QTL were mapped to a resolution of 2 and 3 genes, respectively and, in total, 118 QTL (34%) were mapped to a resolution of 5 genes or less (Fig 2F and Table #8 in https://doi.org/10.5281/zenodo.17381713). For conditions with well-characterized genetics, such as fluconazole-resistance (Fig 2C) and growth on galactose (below), mapped loci included known target genes resolved to the one- or two-gene level (Table #8 in https://doi.org/10.5281/zenodo.17381713). The ability to accurately identify small numbers of genes, including single genes, underlying many QTL demonstrates that CYClones is a powerful tool for producing candidate gene lists highly enriched for causative genes, enabling the biological mechanisms underlying phenotypic variation to be elucidated.

### Heterogeneous allele effects characterize pleiotropic loci

We next leveraged the fact that the ten traits chosen for this study showed very little correlation (Table #7 in https://doi.org/10.5281/zenodo.17381713) and displayed very distinct QTL landscapes (S1 Fig and Tables #2, #3, and #8 in https://doi.org/10.5281/zenodo.17381713), as expected for traits that require distinct biological processes. As such, a causative gene impacting multiple conditions in our study would be consistent with the most conservative definition of pleiotropy, where a single gene impacts multiple distinct processes. We therefore tested whether our ability to map multiple QTL to single gene resolution would allow us to identify cases in which genes exhibited pleiotropic effects. To identify statistically significant instances of pleiotropy, we performed permutation analysis and determined that a gene mapped at single resolution in more than two conditions would be unlikely to occur by chance (p-value<0.001). This analysis identified three such genes: *FLO11* (*MUC1*), *HPF1,* and *WHI2*. *FLO11* encodes a flocculin involved in cell adhesion and biofilm formation and was mapped to single gene resolution in five conditions and at two gene resolution in a further five conditions (ten conditions, total). As colony area is sensitive to colony morphology, the *FLO11* signal may in part reflect its roles in adhesion and colony spreading rather than growth rate alone. *HPF1* encodes a cell wall mannoprotein and was mapped to single gene resolution in seven conditions. Finally, *WHI2* encodes a negative regulator of TORC1 and was mapped to single gene resolution in five conditions, and at three gene resolution in another two conditions (seven conditions, total).

**Fig 3:**
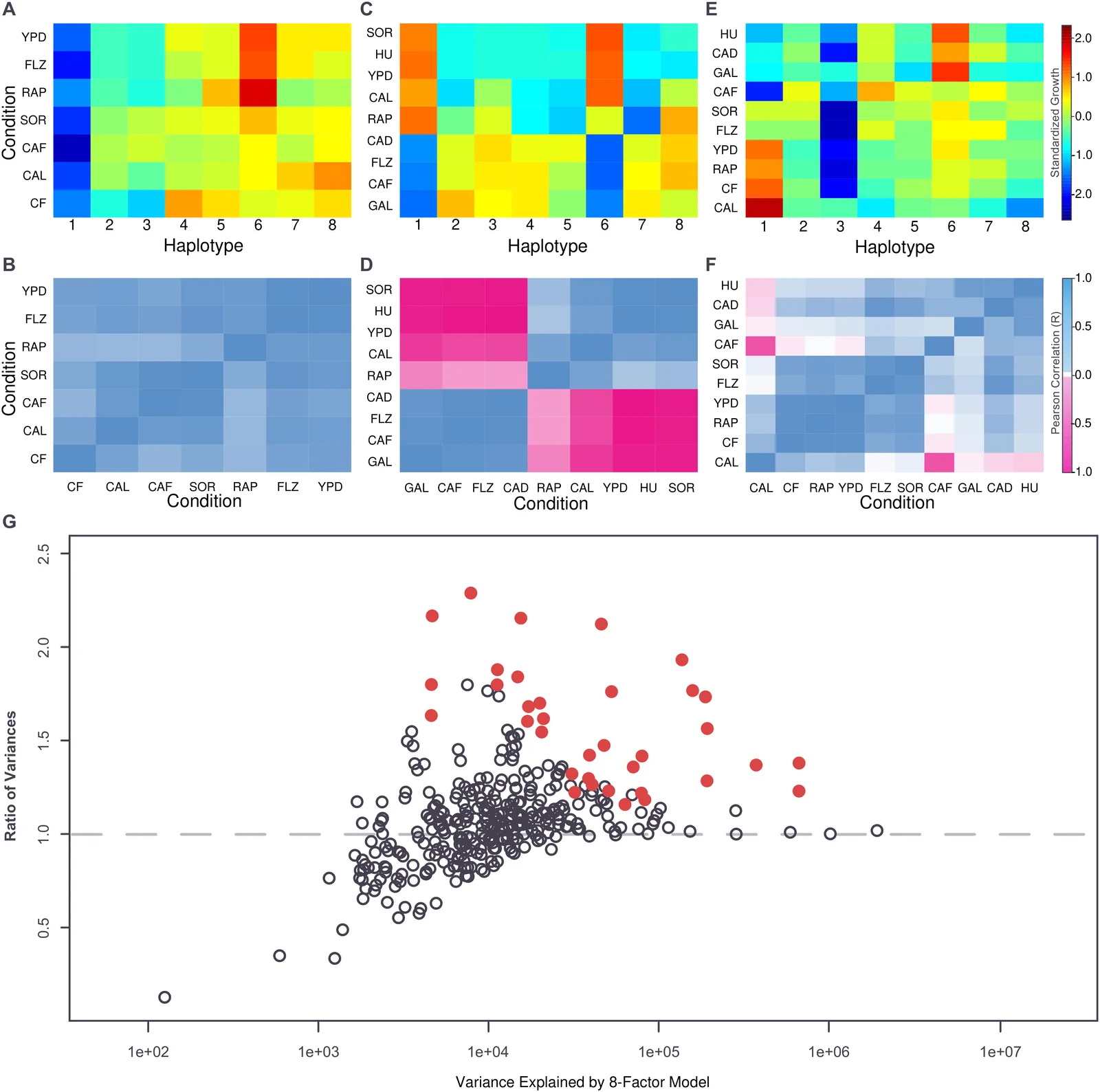
Genetic heterogeneity of QTL and pleiotropic QTL behavior. Standardized haplotype effect sizes (relative to population mean) at *HPF1* **(A)**, *WHI2* (**C**) and *FLO11* **(E)**. Haplotype effect size correlations between conditions for *HPF1* **(B)**, *WHI2* (**D**) and *FLO11* **(F)**. **(G)** Ratio of variance explained by full 8-haplotype model versus binarized model, estimated by cross-validation, for all QTL in additive models for all conditions at 72 **h.** Loci demonstrating significantly (p < 0.05 after Holm correction) better performance of the 8-haplotype model (i.e., polymodal loci) are indicated in red.

Pleiotropy can occur when alleles of a single gene have a consistent effect on multiple traits each having otherwise substantially different underlying genetics (i.e., uncorrelated traits). It can also occur when allele effects at a single locus differ between traits, for example, because multiple variants are present at the locus and these variants each affect different traits. Using our dataset, in the first case, we would expect to see correlated haplotype effects for a pleiotropic locus across different growth conditions. In the second case, we would expect to see uncorrelated haplotype effects. In fact, both patterns are seen across the shared loci identified in our study (Table #9 in https://doi.org/10.5281/zenodo.17381713). For *HPF1*, a single haplotype effect pattern was seen across all conditions (Fig 3A, 3B and Table #9 in https://doi.org/10.5281/zenodo.17381713) with the first principal component explaining 85% of the total variance of the standardized haplotype effects. In contrast, at *FLO11* and *WHI2*, some conditions were closely correlated with each other, while others showed different patterns of haplotype effects. Most dramatically, at *WHI2*, two distinct and opposite haplotype effect patterns were observed (Fig 3C, 3D and Table #9 in https://doi.org/10.5281/zenodo.17381713). In CAL, HU, SOR and YPD, haplotypes 1 and 6 promoted growth relative to the other haplotypes, while in CAD, GAL and RAP these haplotypes inhibited growth relative to the others (Fig 3C and Table #9 in https://doi.org/10.5281/zenodo.17381713). This pattern is consistent with variants at this locus having the same effect on gene activity in all conditions, but in some conditions the gene activity has a positive effect on growth, while in other conditions it has a negative effect.

A more complex pattern of correlations was seen at *FLO11* (Table #9 in https://doi.org/10.5281/zenodo.17381713). At this locus, the variation in effect profiles is driven largely by differing behaviors of haplotypes 1, 3, and 6 (Fig 3E and 3F). The effect of haplotype 1 varies from promoting growth in some conditions, through little effect on growth, to reducing growth (relative to the population mean). Haplotype 3 varies from little effect on growth to reducing growth, while haplotype 6 varies from little effect on growth to promoting growth. The combinations of these effects result in varying degrees of similarity between condition profiles, ranging from the nearly identical profiles seen in CF, RAP and YPD to the strong negative correlation seen between the CAF and CAL conditions. (Fig 3E and 3F). These results are consistent with multiple variants at *FLO11* in our mapping population, distributed differently across the eight founder haplotypes and each impacting the growth conditions in distinct ways, i.e., closely-linked context-dependent causative variants. *FLO11* is known to have a large and complex promoter, bound by a number of transcription factors. Variation at these transcription factor binding sites would be expected to show pleiotropic effects if *FLO11* expression is more strongly impacted by certain transcription factors under certain growth conditions relative to others.

Taken together, these results indicate that a small number of highly pleiotropic loci are observed across the set of uncorrelated phenotypes we examined, but the allele/haplotype effects at each pleiotropic locus can be highly variable across conditions/phenotypes. Multiple closely-linked variants may contribute to this heterogeneity, with effects on phenotype that vary across the ten conditions. Because these closely linked variants would have different levels of fitness across different phenotypes/conditions, evolutionary selection on this kind of locus may be complex, with tradeoffs between the different phenotype/condition-specific fitness effects.

### QTL often reflect multiple, closely linked causative variants

Our pleiotropy analysis suggested that multiple closely linked variants at a single gene could contribute to phenotypic variance across different conditions. We next set out to investigate whether any of the single-condition QTL in our dataset also result from the effect of multiple, closely linked variants rather than the impact of a single variant. The phenomenon where multiple variants at a single locus contribute to phenotypic variation is described as allelic heterogeneity. Even with our highly powered resource, it is not feasible to comprehensively map causative variants to the nucleotide level. We therefore instead searched for statistical signals that are inconsistent with a single variant underlying a QTL. In the mapping population, recombination occurs only rarely between closely-linked variants, with ~0.09 crossovers per 10 kb after 3 rounds of meiosis (described in the companion paper: https://doi.org/10.1101/2025.10.15.682626), so that the parental haplotypes remain mostly intact over distances of <10 kb. This means that the effect of closely-linked variants is difficult to isolate through recombination. However, unlike a pairwise cross where there are only two haplotypes, in a multi-parent cross, there can be as many haplotypes as there are founder strains, which in the case of CYClones is up to eight distinct multi-locus haplotypes. In the case of a single causative (biallelic) variant underlying a QTL, each of these eight haplotypes will always be associated with one of two phenotypic states (bimodality) (S2A Fig), depending on the allele that is present. In the case of multiple-closely linked variants, bimodality can still occur, but the eight haplotypes can also be associated with more than two phenotypic states (polymodality) through imperfect linkage disequilibrium of the variants among the eight founder strains (S2B Fig). Therefore, although genetic heterogeneity can also underlie a bimodal distribution, any QTL in CYClones with a polymodal haplotype phenotype pattern is inconsistent with a single causative biallelic locus.

On this basis, we examined each QTL identified in the additive models for each of our ten conditions and, using cross validation and controlling for the effect of all other QTL, compared the variance explained by fitting phenotype effects for all eight haplotypes, to that explained by using a binary haplotype recoding. The binary recoding was chosen using k-means clustering (k = 2) of the estimated phenotype effects of the eight haplotypes (Materials and Methods). Our results showed that many QTL, including several of the strongest, are essentially bimodal, with little difference between the variance explained by the full eight haplotypes versus the binary recoding (Fig 3G and Table #10 in https://doi.org/10.5281/zenodo.17381713). In contrast, other strong QTL showed a substantial and significant (p-value<0.05 after Holm correction, see Materials and Methods) increase in the variance explained by the eight-haplotype model versus the binary model, consistent with polymodality (Fig 3G and Table #10 in https://doi.org/10.5281/zenodo.17381713). In particular, the pleiotropic *FLO11* locus displayed strong polymodality across several growth conditions, consistent with the idea that multiple closely linked variants are responsible for the differences in *FLO11* allele effect patterns observed between conditions. In total 36/349 (10.3%) of QTL were significantly polymodal and, therefore, QTL arising from the effect of multiple linked loci are relatively common in our dataset. In addition, the polymodal loci represent only a minimum estimate of the number of QTL that result from allelic heterogeneity. As mentioned above, although QTL with polymodal effect patterns can only arise from multiple closely-linked causative variants, closely-linked variants can also produce QTL with bimodal effect patterns. This can occur if the variants have indistinguishable effect sizes (S2C Fig), or if there are only two allele patterns present among the founder haplotypes (S2D Fig).

### Allelic heterogeneity and introgressions define a complex set of non-additive genetic interactions in the galactose pathway

**Fig 4:**
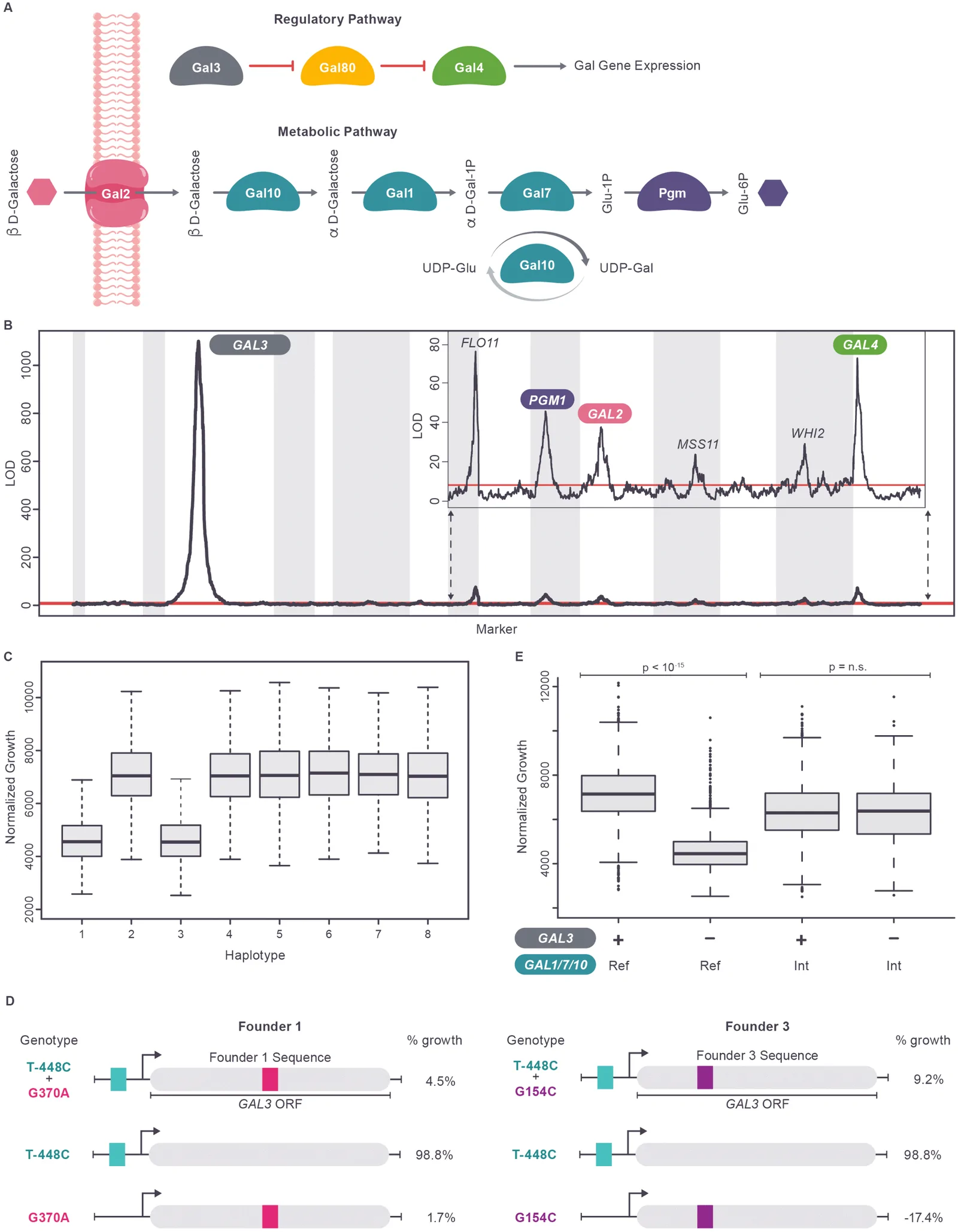
Effect of GAL3 locus on growth on galactose. **(A)** Yeast galactose utilization pathway with genes colored by locus (*GAL1/7/10* form a single locus). **(B)** LOD scores for linkage mapping on galactose. Strong peaks at GAL pathway genes indicated. The *FLO11*, *WHI2* and *MSS11* QTL are not specific to the GAL condition. Insert rescales Y axis to highlight strong QTL other than *GAL3.* 5% family-wise significance threshold in red. Chromosomes alternating in grey and white. **(C)** Growth on galactose for strain subpopulations defined by *GAL3* haplotype. **(D)** Effect on founder 1 and founder 3 growth on galactose after transformation with plasmids expressing their own *GAL3* allele (top) or derivatives (below). Growth is scaled relative to transformation with the S288c reference allele of *GAL3* (100%) and an empty vector (0%). **(E)** Masking epistasis between the *GAL1/7/10* introgression and *GAL3* haplotypes, classified as loss-of-function (*GAL3*-, haplotypes 1&3) or functional (*GAL3* + , remaining haplotypes).

To investigate the statistical signals generated by our QTL analysis in the context of a known biological process, we compared our growth results from the galactose condition to the composition and structure of the well-characterized galactose utilization pathway. The enzymatic components of this pathway consist of the Gal2 galactose transporter and the catalytic enzymes Gal1, Gal7, Gal10, Pgm1, and Pgm2 (Fig 4A). While *GAL1*, *GAL7* and *GAL10* (hereafter *GAL1/7/10*) are a cluster of adjacent genes on chromosome II, the remaining genes are dispersed across the genome. The pathway is transcriptionally regulated by the Gal4 transcriptional activator, whose activity is modulated by Gal3 and Gal80, forming a three-protein regulatory circuit (Fig 4A).

In our dataset, the genetic architecture of the GAL condition is dominated by a single QTL (Fig 4B), which mapped to single gene resolution at *GAL3* and explained 42% of the total variance and 71% of narrow sense heritability (Fig 4B and Tables #3, #8 in https://doi.org/10.5281/zenodo.17381713). The phenotype distributions of the eight *GAL3* haplotype subpopulations are bimodal (Fig 4C), with haplotypes from founders 1 (Tuba wine, Philippines) and 3 (Raffia wine, Nigeria) associated with low growth (*GAL3*^*-*^) and the remaining six haplotypes associated with high growth (*GAL3*^*+*^). Because these results are consistent with a single bi-allelic causative locus, we examined the *GAL3* sequences of the 8 founder strains and identified the only shared variant unique to these two haplotypes, a single SNV (T-448C) in the *GAL3* promoter of founders 1 and 3. To resolve the *GAL3* locus to the quantitative trait nucleotide (QTN), we therefore constructed and assayed substitution mutations that changed this SNV in the promoters of founders of 1 and 3 to the reference genome allele (found in the other six haplotypes). Surprisingly, reverting this shared allele (T-448C) had no effect on the galactose growth of these founder strains (Fig 4D). Instead, we discovered that two different, closely linked, missense alleles in the coding regions of *GAL3,* one unique to founder 1 (c.G370A, p.Gly124Arg) and another unique to founder 3 (G154C/Gly52Arg)*,* were sufficient to confer the loss-of-function phenotype (Fig 4D). That is, the *GAL3* QTL results from two different *GAL3* variants producing the same low-growth phenotype (allelic heterogeneity) rather than from the effect of a single biallelic locus. This is an example of allelic heterogeneity existing even when the phenotypes associated with the eight founder haplotypes are almost perfectly bimodal (see preceding section and Table #10 in https://doi.org/10.5281/zenodo.17381713).

Because genetic variation at *GAL3* has such a strong effect on the GAL phenotype, non-additive pairwise interactions involving *GAL3* also have the potential to significantly impact the phenotype. We therefore scanned for any significant non-additive pairwise interactions between the *GAL3* locus and each of the scaffolding markers. This identified a highly significant interaction between *GAL3* and the *GAL1/7/10* region (S3 Fig), despite there being only a very weak QTL peak detected at the *GAL1/7/10* region alone in the original single marker QTL scan (Table #3 in https://doi.org/10.5281/zenodo.17381713), i.e., *GAL1/7/10* behaves as a *GAL3* modifier locus. Characterizing this interaction in more detail, we observed that in strains having haplotype 6 at the *GAL1/7/10* locus, variation at the major *GAL3* locus had no effect on growth, with *GAL3*^*+*^ and *GAL3*^*-*^ strains displaying the same mean phenotypes (Fig 4E), i.e. full masking epistasis. This striking result underscores that even a major-effect locus like *GAL3* can exhibit strong context dependence, with its phenotypic consequences entirely contingent on variation at interacting loci such as *GAL1/7/10*.

Founder strain 6 (South African soil) has previously described introgressions of non-*cerevisiae* sequences at the *GAL1/7/10* locus (S4A Fig), as well as at *GAL2* and *PGM1* [37]. Our study therefore identifies (p-value = 1.1x10^-149^) a strong non-additive interaction between the presence or absence of the introgression at *GAL1/7/10* and variation at *GAL3*. In contrast, no significant pairwise interaction was observed between *GAL3* and the introgressions at *GAL2* (p = 0.81) and *PGM1* (p = 0.13). A previous study [37] determined that expression of the introgressed genes (in founder 6) is not repressed in glucose, suggesting their expression may have escaped downregulation by the Gal3-Gal80-Gal4 regulatory circuit. Our *GAL3*-introgression interaction results suggest that it is specifically the *GAL1*/7/10 introgression that is responsible for the escape from control by the regulatory circuit.

In addition to these results, our data confirm [37] that strains harboring introgressions at all three loci grow significantly better on galactose than strains with non-introgressed (“native”, i.e., haplotypes 1:5,7,8) alleles (one-sided t-test, p = 3.8x10^-3^) and that combinations of native *PGM1* and the introgressed alleles of *GAL1/7/10* and *GAL2* exhibit significantly lower growth (one-sided t-test, p = 1.0x10^-19^) than strains harboring only native alleles (S4B Fig). This low growth is likely due to the build-up of toxic intermediates caused by combining high-activity introgressed alleles and low activity native alleles at these three loci. Our results extend the previously observed 3-way genetic interaction between introgressed and native alleles at *PGM1*, *GAL1/7/10* and *GAL2* [37] into a significant (p = 7.5x10^-171^) 4-way interaction that includes *GAL3* (*GAL3*^*+*^ vs *GAL3*^*-*^). Notably, both the introgression-carrying founder (founder 6) and a founder with a loss-of-function GAL3 allele (founder 3) were among the parents of the round-robin panel of Bloom et al. [14], the resource used [37] to characterize the *GAL1/7/10*–*GAL2*–*PGM1* introgressions. However, because that panel was built from independent pairwise crosses, each segregant inherits alleles from only two founders, and no single cross combined the introgression with a *GAL3* loss-of-function allele, so their interaction could not be detected. By making every strain a mosaic of all eight founders, the CYClones funnel design reveals such multi-locus interactions that round-robin or biparental designs may not be able to detect. Altogether, these findings underscore the context dependence of genetic effects in the galactose utilization pathway, as the functional impact of specific variants is modulated by the surrounding genetic background, including variation in both the regulatory and structural components of the GAL pathway. In particular, introgressed alleles may alter or bypass native regulatory mechanisms, enabling components of the pathway to escape canonical transcriptional control and thereby reshaping the genetic context in which variants in regulatory genes, such as those at *GAL3*, exert their effects.

### Allele dependent stratification of the galactose pathway: subgroups characterized by distinct regulatory states and genetic architectures

**Fig 5:**
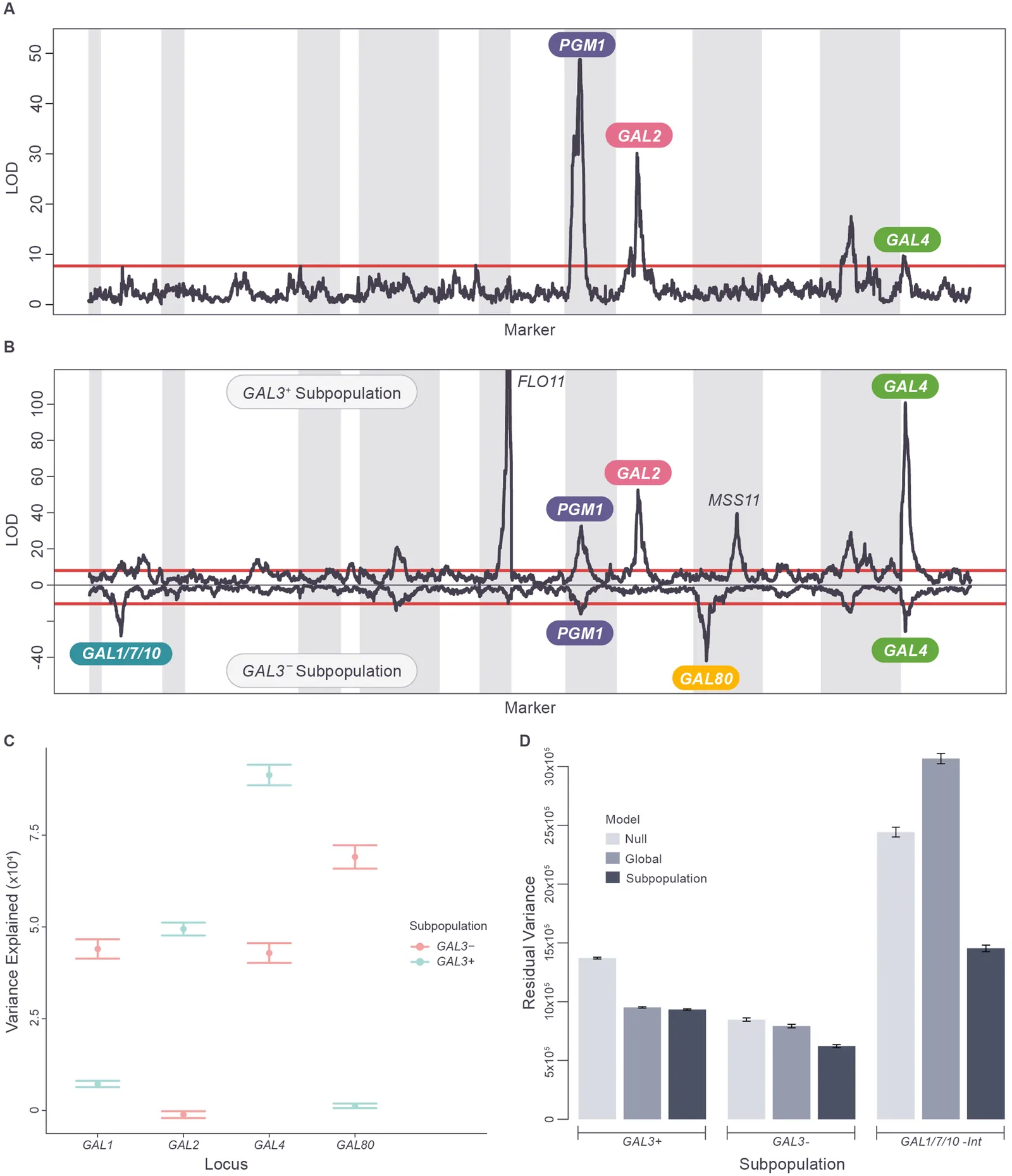
Genetic architecture of growth on galactose. GAL pathway genes labelled when falling in QTL peak 2-LOD confidence intervals, 1% significance thresholds in red. **(A)** QTL mapping in strains with the introgressed *GAL1/7/10* locus. **(B)** QTL mapping in subpopulations lacking the introgressed *GAL1/7/10* locus, split based on *GAL3* + vs *GAL3*-. *GAL3*- LOD scores plotted on negative scale. The *FLO11*, *WHI2* and *MSS11* QTL are not specific to the GAL condition. (**C)** Variance (+/- standard error) explained by individual GAL loci in each subpopulation (in the absence of the *GAL1/7/10* introgression) using random sampling cross validation. **(D)** Performance of global and subpopulation-specific additive models on each subpopulation, assessed by random sub-sampling validation (mean residual estimate + /- SE values shown).

The interaction between *GAL1/7/10* and *GAL3* stratifies the mapping population into subgroups of strains in three different regulatory states. In strains with the *GAL1/7/10* introgression, the effect of variation at the master *GAL3* regulator is suppressed, and variation in growth on galactose is dominated by the interactions between the introgressed and native alleles at *PGM1* and *GAL2* (Figs 5A and S4B and Table #11 in https://doi.org/10.5281/zenodo.17381713). Conversely, in strains without the *GAL1/7/10* introgression, growth is dominated by variation at *GAL3*, with the *GAL3*^*+*^ strains inducing the GAL pathway and promoting growth, relative to the *GAL3*^*-*^ strains (S5 Fig). To determine the effect of these latter two regulatory states on genetic architecture, we therefore carried out QTL mapping in the *GAL3*^*+*^ and *GAL3*^*-*^ subgroups (after removing strains with the *GAL1/7/10* introgression). For clarity, we refer to these sets of strains as ‘subgroups,’ to distinguish them from genetically diverged subpopulations. This approach allows us to assess how the phenotypic effects of genetic variants are shaped by context dependence, that is, how their impact varies depending on the regulatory background established by *GAL3* and *GAL1/7/10*.

The QTL landscapes of the two *GAL3* subgroups displayed substantial differences (Fig 5B and Tables #11, #12 and #13 in https://doi.org/10.5281/zenodo.17381713). First, the effect of variation at QTL containing the regulatory genes *GAL80* and *GAL4* differed between the two subgroups in a manner consistent with the logic of the regulatory circuit (Fig 4A). A significant QTL was seen at the *GAL80* locus (mapped to a 4 kb interval) in the *GAL3*^*-*^ strains, while no significant QTL was observed at *GAL80* in the *GAL3*^*+*^ strains (Fig 5B and 5C and Tables #11, #12 and #13 in https://doi.org/10.5281/zenodo.17381713). Genetic variation in *GAL80* only has a significant phenotypic impact in the presence of the loss of function alleles of *GAL3*, when Gal3 no longer inhibits Gal80 (Fig 4A). Conversely, the phenotypic effect of variation at the *GAL4* locus is significantly stronger (two-sided t-test p = 1.2.x10^-26^) in the *GAL3*^*+*^ subgroup than in the *GAL3*^*-*^ subgroup (Fig 5B and 5C and Tables #11, #12 and #13 in https://doi.org/10.5281/zenodo.17381713), which is also consistent with the structure of the regulatory circuit (Fig 4A), as Gal4 is derepressed in the *GAL3*^*+*^ strains.

In addition to differences in the phenotypic effects of variation in the *GAL4* and *GAL80* regulatory genes, the *GAL3*⁺ and *GAL3^−^* subgroups also displayed different effects of variation at loci involved in mechanistic steps of the galactose utilization pathway (Fig 5B and 5C). There is a significant QTL peak at *GAL2* only in the high-growth subgroup (mapped to an 11 kb interval), while the *GAL2* locus has no significant effect on phenotype in the low-growth subgroup. Conversely, variation at *GAL1/7/10* (introgressed haplotype 6 excluded) had a stronger effect on growth in the low-growth subgroup (two-sided t-test p = 2.8.x10^-25^; Fig 5B and 5C). This differential sensitization to variation at *GAL2* vs *GAL1/7/10* may reflect differences in the degree to which the activity of each protein is limiting when the GAL pathway is induced (*GAL3*^*+*^) vs non-induced (*GAL3*^*-*^).

Taken together, these results illustrate a strong form of context dependence, in which the genetic effects of both regulatory and structural loci shift depending on the regulatory state imposed by *GAL3* and *GAL1/7/10*. The distinct genetic architectures associated with the three identified subgroups of the mapping population (*GAL1/7/10* introgression, *GAL3*^*+*^, *GAL3*^*-*^) mean that a single additive model fitted to the entire population cannot describe the genotype-phenotype relationship of all three subgroups well. In fact, a global model appears to capture the genetic architecture of the *GAL3*^*+*^ subgroup (69% of strains) quite accurately (Fig 5D), reducing residual phenotypic variance significantly relative to a subgroup null model (31% reduction, two-sided t-test p = 1.9.x10^-99^) and performing similarly to a subgroup-specific additive model (32% reduction relative to null model). The global model performs less well (Fig 5D) on the *GAL3*^*-*^ subgroup (22% of strains), reducing residual phenotypic variance significantly (two-sided t-test p = 0.01) relative to a subgroup null model but performing less well than a subgroup-specific model (6% vs 27% reduction in residual variance, two-sided t-test p = 3.1.x10^-15^). On the *GAL1/7/10* introgressed subgroup (9% of strains), the global model performs very poorly (Fig 5D), actually significantly increasing residual phenotypic variation relative to a subgroup null model (28% increase, two-sided t-test p = 2.4.x10^-20^) and performing much worse (two-sided t-test p = 1.2.x10^-71^) than a subgroup-specific model (40% decrease in residual variance relative to null).

It is important to note that in the analyses described in this section, the subgroups analyzed share the same patterns of variation at all loci other than the *GAL1/7/10* and *GAL3* regions used to define the subgroups. Therefore, the subgroups are not genetically diverged subpopulations, instead, they display different genetic architectures despite sharing the same genome-wide patterns of genetic variation, a striking example of context-dependent genetic effects driven by allele-specific regulatory states. We refer to this phenomenon, a form of epistasis where allelic variation determines genetic architecture, as “allele-dependent stratification”. In the case of our GAL results, these distinct architectures appear to result from distinct regulatory states associated with allele state at *GAL1/7/10* and *GAL3*.

### QTL for growth on cadmium chloride: an example of quantitative allele dependent stratification

Our analysis of the galactose growth condition revealed that allele-dependent stratification corresponded to qualitatively distinct regulatory states. Analysis of the major QTL for growth on cadmium chloride extended this qualitative model to characterize a quantitative, allele-determined transition between genetic architectures, a striking example of context dependence, where the functional effects of genetic variants shift continuously with changes in the internal cellular environment.

The strongest single QTL identified in our analysis was observed in the cadmium chloride (CAD) condition and maps to the right end of chromosome II (LOD = 2434; 70% of total variance) (Tables #3 and #8 in https://doi.org/10.5281/zenodo.17381713). Further analysis determined that Founder 2 (United States soil) contains an introgression replacing several genes in this region with orthologs from another species (S6 Fig and File #1 in https://doi.org/10.5281/zenodo.17381713). Reads from this introgressed region align poorly to the budding yeast reference sequence, causing undercounting of haplotype 2. Therefore, to improve sensitivity for identifying strains inheriting introgressed sequences from this region, we re-haplotyped the right end of chromosome II using an introgression-aware pipeline (Methods).

**Fig 6:**
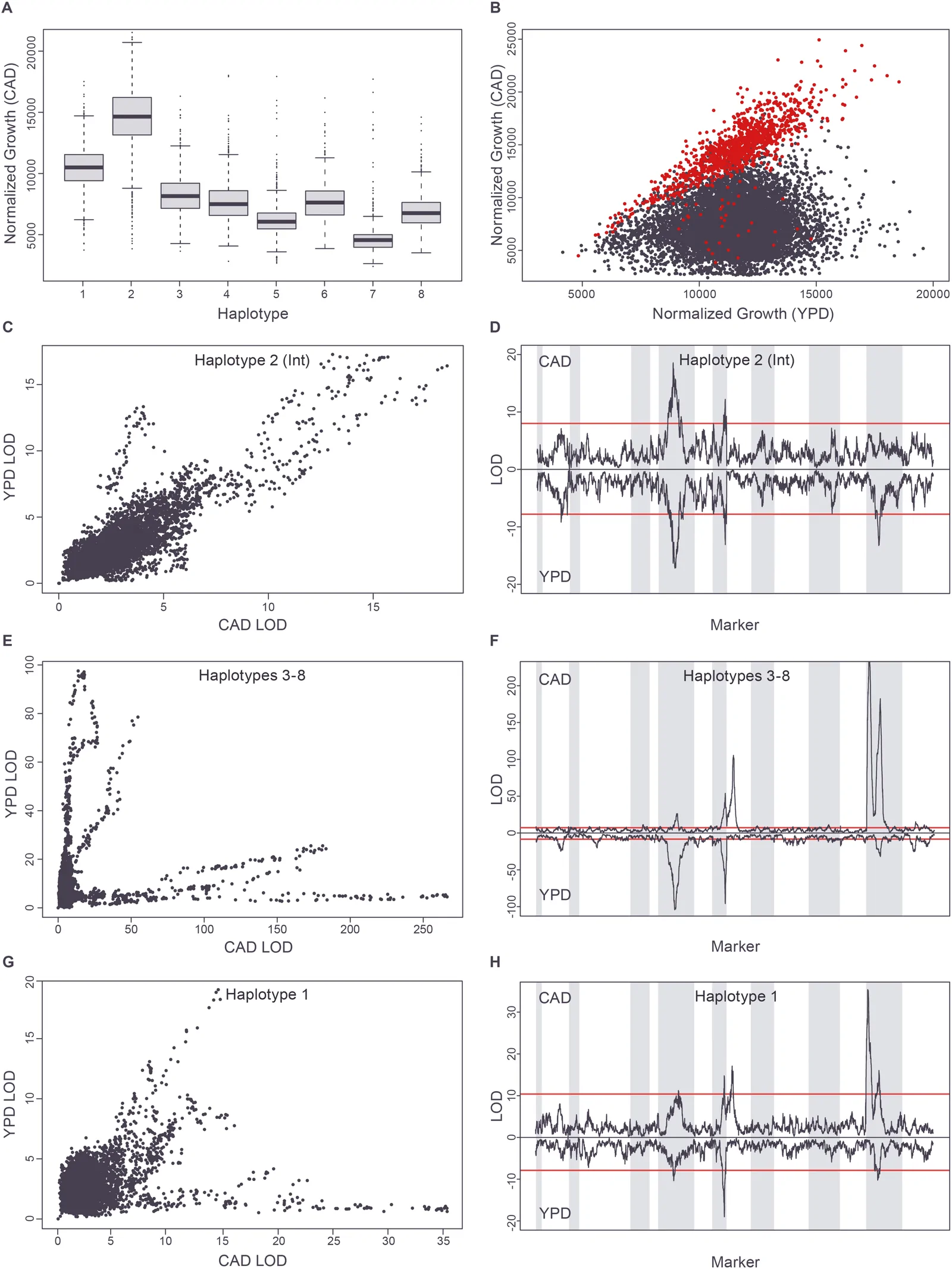
PCA1 haplotypes define a quantitative shift between two genetic architectures on CAD. **(A)** Strain growth on CAD based on *PCA1* haplotype. **(B)** YPD vs CAD growth, strains with the *PCA1* introgression (haplotype 2) in red, rest in black. (**C,E,G**) LOD scores for each scaffolding marker on YPD versus CAD for each of the identified subpopulations defined by *PCA1* haplotype (after accounting for the effect of *PCA1* haplotype for the combined haplotype 3-8 population). (**D,F,H**) LOD scores for each scaffolding marker on CAD and YPD plotted against scaffolding marker number for each of the identified subpopulations defined by *PCA1* haplotype, with YPD LOD scores plotted on a negative scale. Chromosomes indicated as alternating grey and white backgrounds. 1% significance thresholds in red.

After re-haplotyping and performing linkage analysis, the QTL in this region (Table #14 in https://doi.org/10.5281/zenodo.17381713) became even stronger (max LOD = 2624; 72% of total variance), dominating resistance to cadmium chloride with an almost Mendelian genetic architecture and mapping to a 2.4 kb interval (Table #15 in https://doi.org/10.5281/zenodo.17381713) that includes the gene *PCA1*, which encodes a cadmium efflux pump [38]. The effect of the *PCA1* haplotype on growth in CAD was complex. Strains harboring the introgressed *PCA1* haplotype grow much more rapidly than the other strains, but there are also large differences in growth between the seven non-introgressed haplotypes at *PCA1* (Fig 6A), with haplotype 1 growing best and haplotype 7 growing worst. This strongly suggests that multiple *PCA1* variants are impacting function (allelic heterogeneity), and that these variants are in linkage disequilibrium among the seven (non-introgressed) founder strains.

For the entire mapping population, growth in the presence (CAD condition) and absence (YPD condition) of cadmium is essentially uncorrelated (R^2^ = 0.029), although growth on YPD does define an upper boundary for growth on CAD (Fig 6B). However, strains with the introgressed *PCA1* haplotype grow so well on CAD (Fig 6A) that we hypothesized they might behave as if they were growing in the absence of this heavy metal stressor. Indeed, growth rates on CAD and YPD are strongly and significantly correlated among strains carrying the introgressed *PCA1* gene (R^2^ = 0.61; p-value = 5.9x10^-248^), and these strains fall on the upper boundary of growth on CAD (Figs 6B and S7). In addition, the QTL profile of the introgressed strains on CAD and YPD is almost identical (Fig 6C and 6D and Table #16 in https://doi.org/10.5281/zenodo.17381713). These results are consistent with the *PCA1* introgression making strains fully resistant to cadmium, so that their growth is constrained by the same genetics that determine their growth in the absence of cadmium (i.e., on YPD).

In contrast to the introgressed strains, strains having haplotypes 3–8 at the *PCA1* locus show lower levels of resistance to cadmium (Fig 6A) and display low correlation between growth on YPD and CAD (R^2^ < 0.085 for every haplotype), (S7 Fig). Similarly, the genetics determining growth on YPD and CAD differ strongly for these strains (Fig 6E and 6F). On YPD, the QTL trace of the combined haplotype 3–8 strains is very similar to that seen for strains with the *PCA1* introgression (Fig 6D and 6F and Table #16 in https://doi.org/10.5281/zenodo.17381713, R^2^ = 0.64), representing a shared YPD genetic architecture. On CAD, after accounting for the effect of *PCA1*, haplotypes 3–8 share a single genetic architecture (S8 Fig) with the first principal component explaining 87% of standardized variance in their LOD profiles. This architecture is very distinct from the common YPD architecture and includes major peaks including the cadmium-related genes *SMF1*, a transporter involved in the transport of cadmium and other divalent ions (mapped to a 3.4 kb interval), and *GSH1*, a glutathione biosynthesis gene involved in the cellular response to cadmium [39] (mapped at single gene resolution to a 1.5 kb interval) (Table #17 in https://doi.org/10.5281/zenodo.17381713). Therefore, among strains with these six *PCA1* haplotypes (haplotypes 3–8), the degree of growth on cadmium, after accounting for the effect of the major *PCA1* locus, appears to be largely determined by additional loci specifically affecting cadmium resistance rather than the genetics determining growth on YPD.

The final set of strains, with haplotype 1 at the *PCA1* locus, shows the second highest mean level of growth on cadmium (Fig 6A). Like the introgressed subgroup, these strains display a correlation between growth on cadmium chloride and on YPD, but lower than that seen with the introgressed strains (R^2^ = 0.28 vs 0.61; S7 Fig). On YPD, the haplotype 1 strains display the same genetic architecture seen in the other *PCA1* subgroups (Fig 3H and Table #16 in https://doi.org/10.5281/zenodo.17381713), while the QTL landscape of these strains on CAD is a mixture of the cadmium resistance architecture seen with haplotypes 3–8 and the YPD-like architecture seen in the introgressed strains (Fig 6G and 6H and Table #16 in https://doi.org/10.5281/zenodo.17381713). These results are consistent with a quantitative genetic transition between growth constraint by cadmium chloride and growth constraint by YPD, a form of context dependence in which the genetic architecture is modulated by the physiological consequences of allelic variation at *PCA1*. Because Pca1 exports cadmium ions from the cell, the CAD-specific and YPD-like constraint regimes associated with the different *PCA1* alleles may reflect varying levels of toxic intracellular cadmium associated with different levels of Pca1 activity. In this model, *PCA1* haplotypes 3–8 result in relatively low levels of Pca1 activity, leading to high levels of toxic intracellular cadmium and a cadmium-specific genetic architecture constraining cell growth. In contrast, *PCA1* haplotype 2 results in relatively high export activity, leading to low levels of intracellular cadmium and cells with a YPD-like genetic architecture constraining their growth. Finally, *PCA1* haplotype 1 leads to intermediate levels of export activity and intermediate levels of intracellular cadmium, placing strains with this allele in the transitional zone between the two constraint regimes and producing a mixed genetic architecture.

## Discussion

### Overview and context

The CYClones multiparent population provides a powerful resource for dissecting the genetic architecture of complex traits, combining large numbers of mapping strains, dense recombination and individual-strain haplotyping. Here we show that, combined with accurate high-throughput phenotyping, CYClones maps many QTL across diverse conditions, frequently resolving them to the level of single genes or small candidate gene sets. Beyond detecting and localizing loci, its scale and resolution let us characterize features of genetic architecture that are usually difficult to resolve: the wide spectrum of trait complexity, pervasive context-dependent effects, widespread allelic heterogeneity, the form of epistasis we term allele-dependent stratification, and pleiotropy among a subset of loci. Below, we discuss how CYClones relates to existing cross-based and natural-population resources, before turning to consider these findings.

Approaches to yeast QTL mapping fall along a continuum that trades the breadth of natural variation sampled against the power and resolution achievable per variant. At one end, biparental crosses such as BY × RM [17,18], and X-QTL pooled-segregant experiments [19], each locus carries only two alleles at ~50% frequency, maximizing the power to detect a given variant for a fixed number of strains. This advantage has been pushed to its limit in two directions: barcoded bulk QTL mapping has been used to genotype ~100,000 segregants from a single cross to capture very-small-effect, highly polygenic contributions [20], while the single-nucleotide mapping approach of She and Jarosz [40] crosses closely related strains so that dense recombination resolves the few segregating variants to the causal change. Both, however, share the limitation intrinsic to a single cross: with only two alleles per locus, they cannot access the variants and allelic series carried by other genetic backgrounds.

At the other end of the continuum, natural-population genomics and GWAS [41,42] and diallel hybrid panels [25,26] sample the full species-wide allelic spectrum, far exceeding any cross. This breadth comes with costs, however: confounding from population structure and clonal-history linkage disequilibrium, and limited power for rare alleles.

Multiparent populations such as CYClones occupy the middle ground, capturing much of the advantage of each pole while avoiding its principal weakness. Relative to GWAS, a controlled pedigree and known founder genomes remove the confounding from population structure and clonal linkage disequilibrium and bring rare founder variants to mappable frequency, at the cost of sampling fewer alleles than the species-wide spectrum. Relative to biparental crosses, multiple founders broaden allelic coverage and, critically, enable the detection of interactions among alleles contributed by different founders, such as the four-locus GAL interaction described above, that pairwise designs are less likely to assemble, while retaining substantial power and resolution.

Among existing yeast multiparent resources, CYClones is further distinguished by its design. The four-parent SGRP-4X [21] samples fewer founders; the 16-founder Bloom et al. panel [14] and the 12-founder panel of Treusch et al. [22] are round-robins of pairwise crosses, so each segregant descends from only two founders; and the 18-way Linder et al. populations [23] rely on pooled genotyping of a long-outcrossed diploid pool. By contrast, the CYClones funnel makes every haploid strain an individually genotyped eight-founder mosaic, combining competitive power, single-gene resolution, and access to multi-founder allelic series and interactions in one resource.

Across this landscape of biparental crosses, other multiparent panels, and natural-population GWAS, no single design dominates; each balances power, resolution, and allelic breadth differently, and the approaches are best regarded as complementary. CYClones occupies a distinctive niche within this landscape, combining the mapping power and single-gene resolution of a controlled cross with access to multi-founder allelic series and the context-dependent interactions that only a multi-founder pedigree can resolve. It is precisely these context-dependent effects, and the broader diversity of genetic architectures they produce, that the CYClones dataset brings into focus across the ten conditions examined here.

### Genetic complexity in the architecture of quantitative traits

One of the most striking features revealed by the CYClones resource is the diversity and richness of genetic architectures underlying the quantitative traits examined. Some traits are dominated by a single, large-effect locus, while others exhibit highly polygenic architectures shaped by many small-effect variants. This variability arises not only from differences in effect size and number of contributing loci, but also from other components of genetic architecture, including pleiotropy, allelic heterogeneity, and epistatic interactions, that modulate phenotypes in condition-specific ways.

Although most loci affected growth in a condition-specific manner, we identified a small number of broadly pleiotropic loci that influenced multiple traits. Notably, *WHI2*, *FLO11*, and *HPF1* each affected growth in at least seven conditions, often with trait-specific effect directions and magnitudes. For instance, at *WHI2* and *FLO11*, the same haplotype promotes growth in one condition while reducing it in another, suggesting that the same functional allele can have opposing fitness consequences depending on cellular context. These findings illustrate that even when pleiotropy is limited to a few loci, it can introduce substantial complexity and asymmetry into genetic architectures. We note that pleiotropy is likely more widespread than the three loci we examined, as we only considered QTL mapped to single-gene resolution. Moreover, the opportunity to identify pleiotropy was limited by the ten specific growth conditions tested, so the QTL we identified may affect many other phenotypes. Indeed, CYClones will become increasingly powerful for understanding genotype–phenotype relationships as additional traits are measured, particularly given that the mapping population and all sequencing and genotyping data are broadly available to the research community.

Our analysis also reveals that allelic heterogeneity is common, with many QTL best explained by the combined effects of multiple, tightly linked causal variants. Across the genome, 10.3% of QTL exhibit significantly polymodal haplotype effect patterns, inconsistent with a single biallelic variant and indicative of multiple functional alleles contributing distinct phenotypic effects. In the galactose condition, we demonstrate this directly at the *GAL3* locus, where two distinct missense mutations in different founder backgrounds independently produce a loss-of-function phenotype. Moreover, *GAL3* interacts epistatically with introgressed alleles at the *GAL1/7/10* locus, and these interactions, along with additional non-additive effects involving introgressions at *GAL2* and *PGM1*, form a complex, multi-locus regulatory network. The phenotypic consequences of these interactions depend on regulatory state and specific combinations of introgressed and native alleles, illustrating how both allelic heterogeneity and epistasis can structure genetic architecture at the pathway level.

Introgression of alleles from other yeast species is a key contributor to the complexity of genetic architectures that we observed. In both the cadmium (CAD) and galactose (GAL) conditions, introgressed alleles reshape cellular physiology in ways that radically alter the genetic architecture of the trait. These introgressed sequences do not act in isolation but interact with native loci, generating condition-specific architectures that defy simplistic additive models. As such, introgression not only contributes to phenotypic variance but also increases architectural diversity across traits. These results provide important context for evaluating the molecular consequences of introgression in more complicated organisms, including Neanderthal and Denisovan introgressed sequences in modern humans [43,44].

More broadly, these findings emphasize that genetic complexity is not merely a matter of polygenicity. Instead, it encompasses multiple overlapping phenomena, including pleiotropy, allelic heterogeneity, introgression, and context-dependent interactions, that interact to produce rich, condition-specific genotype-phenotype maps. These interlocking layers of complexity highlight the need to understand genetic architecture not just as a static map of effect sizes, but as a dynamic system shaped by regulatory and physiological state.

### Allele-dependent stratification: a specific manifestation of epistasis

Beyond identifying individual loci and their interactions, CYClones allowed us to characterize allele-dependent stratification, a specific manifestation of epistasis in which specific alleles at key regulatory or structural loci effectively partition the population into subgroups that experience distinct genotype-phenotype relationships. This phenomenon differs from the traditional notion of “background effects,” such as those commonly described in mouse models, where allelic effects shift subtly depending on broader genomic context. Instead, allele-dependent stratification arises when specific alleles of a small number of genes produce discrete regulatory or physiological states that, in turn, reshape the genetic architecture of the phenotype itself. Conditional analyses that recover background-specific QTL by conditioning on a major-effect locus are well established (example in [45]); here this occurs at a scale where a single allele acts as a hub for many such loci, reorganizing the architecture across subgroups.

In the GAL condition, the *GAL3* and *GAL1/7/10* loci define three such regulatory subgroups, each with distinct genetic architectures. Similarly, in the CAD condition, variation at the gene encoding the Pca1 efflux pump modulates intracellular cadmium levels, effectively stratifying the population into cells that differ in their physiological exposure to cadmium stress. These differences in intracellular cadmium levels then determine whether genetic variation influencing detoxification pathways or basal growth rate becomes phenotypically relevant. Crucially, in both examples, the genome-wide patterns of variation are shared across subgroups; only the alleles at a small number of loci differ. The resulting architectural differences are therefore not a consequence of population structure or long-term divergence but rather emerge from functionally mediated, allele-specific shifts in internal state, challenging the standard assumption that a single additive model can capture trait variance across a population and underscoring the need for approaches that can incorporate cellular and regulatory state into predictive frameworks.

When faced with such allele-dependent stratification, our results from the GAL condition demonstrate that global additive models default to accurately reflecting the genetic architecture of the largest subgroups while performing poorly on smaller subgroups, whose distinct genetic architectures are not captured in the model. This behavior also explains why allowing non-additive interactions within whole-population models provides little improvement in global performance. Because the model is dominated by the majority subgroup’s architecture, adding interaction terms cannot recover the fundamentally different genetic architectures of the minority subgroups.

It is important to note that allele-dependent stratification is equivalent to a locus acting as a hub of non-additive interactions. These are not distinct phenomena but two descriptions of the same genetic structure: a locus whose allele determines which other loci shape the phenotype is, by definition, a locus that interacts with each of those loci. Because the two are equivalent, the more parsimonious description is preferable: when these interactions are coordinated by a small number of state-defining loci, the full web of pairwise non-additivity collapses into a few subgroups occupying meaningfully distinct regulatory or physiological states. To identify such subgroups, however, is not necessarily straightforward. While one could in principle attempt to comprehensively stratify a population based on allelic variation at each QTL to identify subgroups with distinct genetic architectures, such exhaustive approaches suffer from the same statistical limitations, namely, power loss and multiple-testing burden, that constrain genome-wide interaction analyses. Instead, because these subgroups correspond to distinct regulatory or physiological states, data that report directly on cellular state, such as gene expression, chromatin accessibility, or protein abundance, potentially offer a more direct route to identifying them than genetic stratification alone. Such measurements can reduce the search space and enable targeted modeling of genotype-phenotype relationships within contextually defined strata.

### Implications for human genetic studies

Our findings suggest that allele-dependent stratification may be a general feature of complex trait architecture, with important implications for human genetics and precision medicine. In particular, the presence of a strong disease-risk or protective allele may place individuals into distinct regulatory or physiological states that fundamentally alter the genetic architecture influencing phenotype. For example, individuals carrying loss of function alleles in *CFTR* (leading to cystic fibrosis) or in *PCSK9* (reducing LDL cholesterol and protecting against heart disease) [46] may exhibit different sets of modifying loci than those relevant to individuals without the major effect variant. That is, the genetic modifiers of phenotype may differ depending on whether an individual carries a major-effect allele, such that global polygenic risk models may systematically misestimate risk for subgroups carrying such alleles. While our yeast studies provide proof-of-principle for this phenomenon, these human analogs represent testable hypotheses that warrant investigation.

This principle may extend beyond specific monogenic variants with extreme phenotypes to individuals who fall at the extremes of polygenic trait distributions, those with exceptionally high or low liability scores. At the tails, the physiological or regulatory landscape may be sufficiently perturbed that the relevant genetic modifiers differ from those shaping phenotypes in the center of the distribution. In this way, polygenic extremity itself may define one or more stratified subgroups, with distinct genetic architectures. As a consequence, polygenic risk scores may be particularly prone to misestimating risk in individuals at the extremes, where the underlying assumptions of a uniform genetic architecture no longer hold. Identifying such allele- or state-defined subgroups, through orthogonal data such as transcriptomic or proteomic profiles, could greatly improve the precision and interpretability of risk modeling efforts.

## Supporting information

S1 Fig LOD traces for all conditions at 72 h. Scaffolding marker set used. Chromosomes indicated by alternating grey and white backgrounds. Red line represents 5% family-wise significance threshold.
(TIF)

S2 Fig Patterns of haplotype effect sizes at loci with single causative variants or closely linked pairs of causative variants. (A) A single causative variant site always produces a bimodal effect distribution. (B) Two closely linked causative variant sites permuted among founder haplotypes can produce a polymodal haplotype effect distribution (e.g., G = -2, C=+8, A = -12, T = 0). (C) Two closely linked causative variant sites permuted among founder haplotypes can produce a bimodal haplotype effect distribution if multiple allele permutations share the same effect size. (D) Two closely linked variant sites that are not permuted among founder haplotypes produce a bimodal effect distribution.
(TIF)

S3 Fig Interaction vs additive LOD trace for *GAL3* maximum marker (chr04_465458) vs all scaffolding markers identifies a strong interaction between *GAL3* and *GAL1/7/10.* Chromosomes indicated by alternating grey and white backgrounds. Red line represents 0.1% FDR threshold for full GAL interaction vs additive dataset.
(PDF)

S4 Fig Interactions between reference and introgressed GAL pathway genes. (A) An introgression replaces the *GAL7*, *GAL10* and *GAL1* genes with non-native versions in founder 6. (B) Effect on strain growth of combining introgressed and non-introgressed (reference) alleles of GAL pathway genes.
(TIF)

S5 Fig Effect of *GAL3* locus on growth on galactose for strains lacking the *GAL1/7/10* introgression. (A) LOD scores for linkage mapping on galactose. Chromosomes alternating in grey and white. 1% significance threshold in red. (B) Growth on galactose for strain subpopulations defined by *GAL3* haplotype.
(TIF)

S6 Fig Founder 2 possesses an introgression on the right telomeric region of chromosome II that includes *PCA1.* (A) Neighbor joining tree of *PCA1* protein sequences. (B) The introgressed telomeric region of chromosome II from founder 2 (numbering from File #1 in https://doi.org/10.5281/zenodo.17381713) compared to the S288c reference. All ORFs > 500 bp displayed, with reference homolog names.
(TIF)

S7 Fig Comparisons between growth on YPD and CAD for strains stratified by *PCA1* haplotype. Full distribution of all strains shown in each plot, with current subset of strains indicated in red.
(TIF)

S8 Fig Shared Genetic Architecture on CAD for strains with *PCA1* haplotypes 3–8. LOD scores calculated at each scaffolding marker in each subpopulation plotted against first principal component of the subpopulation LODs (each scaled to unit variance).
(TIF)

S9 Fig Phenotypic distributions of the ten growth traits across the mapping population. Histograms of normalized colony area (patch area in pixels) at 72 h for each of the 9,344 euploid strains, plotted separately for each growth condition. For each strain, the value shown is the mean of the two independent replicate measurements.
(PDF)

S1 Text Supplementary methods. Detailed computational and procedural descriptions providing additional information to support the methods summarized in the main text.
(DOCX)
