# Supplementary material for: Pervasive context-dependent effects in the genetic architecture of complex and quantitative traits revealed by a powerful multiparent mapping population in yeast
Source: PLoS Genet. 2026 Aug 27;22(8):e1012288. doi: 10.1371/journal.pgen.1012288 (PMC13577571; doi:10.1371/journal.pgen.1012288)
Supplement: Supplementary file 1 [file pgen.1012288.s003.pdf]

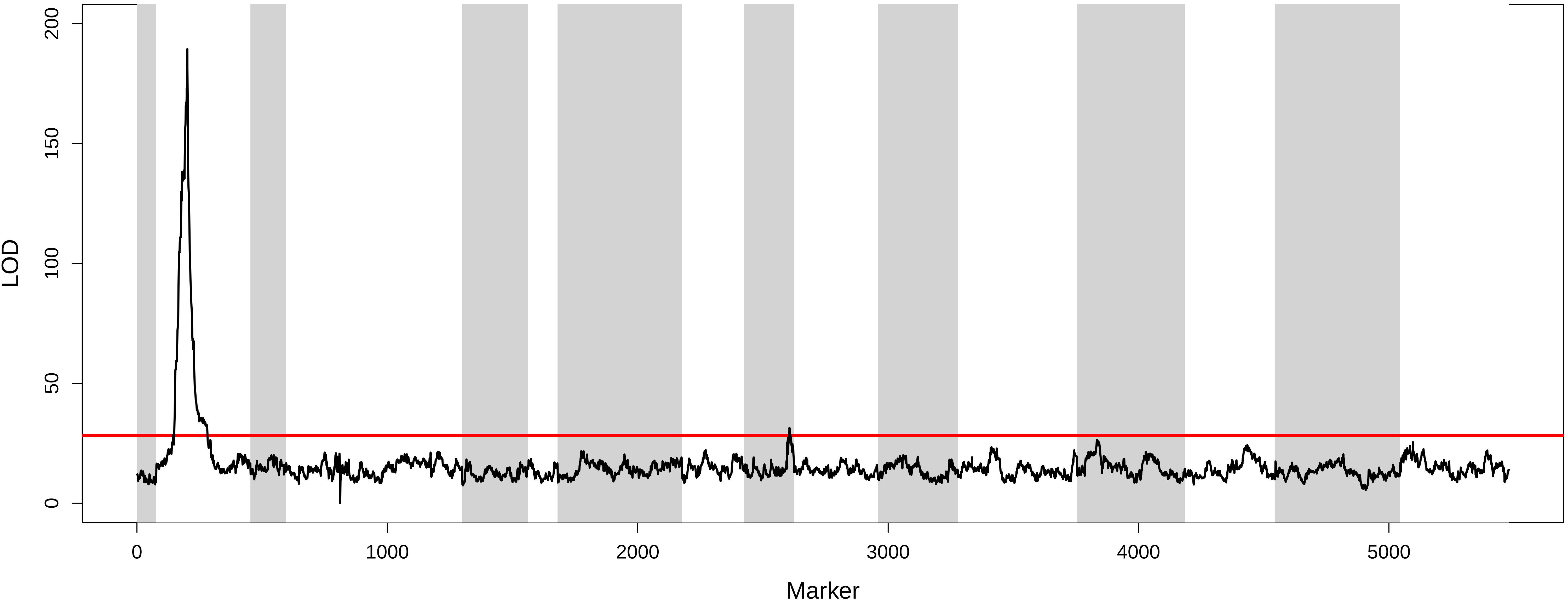

LOD
0
50
100
150
200
0
1000
2000
3000
4000
5000
Marker
