# Supplementary material for: Pervasive context-dependent effects in the genetic architecture of complex and quantitative traits revealed by a powerful multiparent mapping population in yeast
Source: PLoS Genet. 2026 Aug 27;22(8):e1012288. doi: 10.1371/journal.pgen.1012288 (PMC13577571; doi:10.1371/journal.pgen.1012288)
Supplement: Supplementary file 3 [file pgen.1012288.s010.docx]

# S1 Text. Supplementary Methods

These supplementary methods provide detailed computational and procedural descriptions supporting the information in the main Materials and Methods. Citations and table/file references use the same numbering as the main manuscript.

## Replica pinning and image analysis

To generate the source plates for replica pinning, the 96-well glycerol stock plates were thawed and 2.5 µl of each well was transferred to a fresh 96-well source plate containing 150 µl YPD broth per well. A control plate (used to estimate the effect of well position on patch growth) was inoculated with strain YO2685 (founder 1) in all 96 wells. Inoculated source plates were covered with a breathable seal (Agygen, BF-400-S) and incubated at 30°C for 48 hours, without shaking. Following incubation, breathable seals were removed, and cells were resuspended by placing the plates in an orbital shaker at 900 rpm for two minutes. A Biomek i7 (Beckman Coulter) robot fitted with a 96-prong pinner (V & P Scientific, FP6 pins) was then used to spot strains from each source plate to one agar destination plate per experimental condition, shaking the source plate for five seconds between transfers. Once dry, pinned agar destination plates were placed in a 30°C incubator for 72 hours. Plates were briefly removed from the incubator and photographed every 24 hours using a mounted Canon PowerShot SX10 IS digital camera under consistent lighting, camera-to-subject distance, and zoom. Images (ISO200, f4.5, 1/40 s exposure) were acquired as JPEG files. The area (in pixels) and average grey scale value of each replica-pinned patch was extracted with PyPlate, a custom python pipeline built with functions from the scikit-image library (https://github.com/lacyk3/pnri-projects/tree/Image-Analysis-Demos/Funnel%20Cross%20Project).

Using PyPlate, each plate image was cropped into 96 regions of interest (ROIs) centered at the pin locations, with each region containing a single patch. Patch area was measured by segmenting each ROI using a combination of intensity thresholding and circle detection. Intensity thresholding was performed by binarizing the image with the Otsu threshold [27] and applying the morphological operations of closing and filling to segment the bright patch from the dark background. If the threshold returned by Otsu’s method was sufficiently different from the average background agar intensity, the results of Otsu thresholding were considered meaningful. Circle detection was performed by binarizing the image with an adjusted mean threshold and applying the Circular Hough transform [28] with a minimum radius equal to the initial pin radius. This exploits the geometry of the pinned cells to capture any regions of patches where growth is too faint to be detected with intensity thresholding. If the results of the intensity threshold were considered significant, these were combined with the detected circle to form the patch mask. However, if the Otsu threshold was similar to the background agar intensity, then the intensity thresholding was discarded and the detected circle alone was used as the patch mask. An additional check was performed to rule out unreliable results which may have arisen from a blank or contaminated ROI. Specifically, if the mean intensity of the masked patch area was too low or the likelihood of the detected circle was too low, then the patch was determined to be empty and assigned a value of NA. The final patch area is simply the total number of pixels in the patch mask.

## Phenotype data processing and normalization

For 9,329 strains (99.8%) we obtained growth data from both replicates. Data was obtained from a single replicate for 15 strains. The 7 strains for which phenotyping failed in both replicates were removed from further consideration. The growth data were then normalized.

First, area measurements <=2000 pixels (approximate pin deposition area) were replaced with NA values. Along with patches that the image processing pipeline failed to identify, this left 0.3% of measurements as missing data (NA calls). Plate-level normalization was then carried out assuming a multiplicative plate effect, estimated using the 8 founder strain control patches on each plate. After this, multiplicative batch level normalization was carried out, where batches are groups of experimental plates processed in the same week. This normalization utilized all strains, equalizing the median area value for each timepoint + condition across batches. Finally, multiplicative normalization for growth on the four plate edges was carried out assuming a single timepoint-specific multiplier for each edge, adjusted by a condition-specific multiplier (across all edges) for each condition at each timepoint. Final phenotypes were calculated as the mean of all normalized measurements for each strain on each condition at each timepoint (Table #2 in https://doi.org/10.5281/zenodo.17381713).

After these steps, the final dataset included 9,344 haploid (and euploid) strains haplotyped at 276,774 SNP positions and with normalized growth measurements at three time points (24, 48 and 72 hours) across 10 media conditions.

## Fine mapping and cross-timepoint refinement of QTL

We fine-mapped the position of each QTL and generated confidence intervals of the location of each QTL as follows. We started with the additive models built using the scaffolding markers across each timepoint and condition and then, for each marker in each model, that marker was removed from the model and the residuals of the model were adjusted to reverse the phenotypic effects attributed to that marker. Then, linkage mapping using the adjusted residuals was carried on the same chromosome as the “dropped” marker but using all markers (from the full set of ~270k), not just markers from the scaffolding set (~5.5k). The strongest marker was then identified along with a 2-LOD confidence interval. Finally, genes were identified as falling within the confidence interval of each QTL if the interval overlapped a region extending from 1kb upstream to 0.5kb downstream of a gene’s open reading frame.

QTL traces were highly similar across the three timepoints of our experiments (Table #3 in https://doi.org/10.5281/zenodo.17381713): the number of detected QTL was nearly identical (345, 339, and 349 at 24, 48, and 72 h), and the genome-wide rank order of marker LOD scores was strongly concordant between the adjacent 48 h and 72 h timepoints (Spearman ρ = 0.96–0.99; ρ = 0.67–0.92 between the more widely separated 24 h and 72 h timepoints). Based on this, the confidence intervals of the 24h and 48h QTL sets for each condition were used to further refine the gene-level resolution of the QTL measured at the 72h timepoint. For each 72h QTL, the list of genes falling within its 72h confidence interval was compared to the list of genes falling within any QTL confidence interval in the 24h and 48h datasets for the same condition. Only QTL with confidence intervals <100 kb were refined or used for refinement. The genes with the highest (or co-highest) count were retained. Because the set of genes lying within the set of QTL confidence intervals is always a small subset of all genes, this approach effectively examines overlap between confidence intervals for the same QTL at multiple timepoints without having to first estimate the true physical position of the (shared) QTL.

## Variance explained by additive and interaction models

The variance explained by additive models from each timepoint and condition was calculated by 10-fold cross-validation. In each fold, the training data was used to generate an additive model by forward selection, as described (without fine mapping), and the variance explained by that model was calculated on the test data. The mean test-population variance explained across the 10-folds was then calculated.

To examine genetic architecture, we then took the models from each training fold and ordered the markers from the most to the least significant (type II ANOVA p-value) based on the training fit. Next, in each fold, we fit a new model using only the single strongest marker from the training data, measured the variance explained in the corresponding test fold, and took the mean variance explained across all folds. This number represents the variance explained by the strongest single marker model. We then repeated this using the strongest two markers from the training models. This process was continued until the number of markers reached the size of the smallest full model in any of the training folds.

We also took each of the full additive models from the 10 folds and introduced significant non-additive interactions between the loci, in a stepwise fashion. In each training fold, for each pair of QTL, a new model was constructed identical to the additive model except for allowing a non-additive interaction between the two current loci. Each of these models was compared to the base additive model by ANOVA, and ranked based on the resulting p-value. The additive model was then extended, one pairwise interaction at a time, using the rank-ordered pairwise interactions (strongest to weakest). After each model update, the new model was compared to the previous iteration by ANOVA and the process was stopped if the improvement was not significant (p-value<0.001). The average variance explained by the final models was calculated on the corresponding test folds as above.

## Determination of QTL multimodality

For each 72h QTL identified in the fine-mapping additive models, the 8 haplotype effect size estimates were extracted. Estimates associated with haplotypes seen in <50 strains were replaced by NA values. K-means clustering (k=2) was applied to the effect size estimates for each QTL in order to binarize the haplotypes. Then, for each QTL, 100 random 90:10 training:test strain splits were generated. For each training set, three models were fit, the first using the full haplotype encoding at the current QTL (full), the second the binary recoding of the current QTL (binary), and the third lacking the current QTL (null). Each model incorporated (using the original haplotype encoding) all other QTL in the fine-mapping additive model. For each test set, the additional variance explained (relative to the null model) was then calculated for the full and binary models. The distributions of these values were used to calculate whether the full models explained significantly more variance than the binary models (one-sided t-test, p-value<0.05 after Holm correction for all QTL tested across all conditions). The means of the distributions were used to estimate the variance explained by the full and binary models for each marker.

## Identification of pleiotropic genes

To identify QTL mapped to single gene resolution affecting more traits than expected by chance, the number of the single genes mapped in each condition was counted and the identity of these genes was randomized (across all yeast genes) and the maximum number of conditions sharing a gene was calculated for each permutation of all conditions. A total of 10,000 permutations was carried out and the 95% quantile of this null distribution was identified as 2 conditions, so that a gene shared by 3 or more conditions is significant at a p-value<0.05

## GAL statistical and subpopulation analyses

The interaction versus additive model values for each scaffolding marker in combination with GAL3 were taken from the full pairwise interaction versus additive model matrix, using the row corresponding to the peak of the GAL3 QTL, marker chr04_465458.

Because of the strength of the GAL3 QTL, for interaction and subpopulation analyses, strains with crossovers between the markers flanking GAL3 (“chr04_463411” and “chr04_465458”) were removed before further analysis, as their GAL3 QTL haplotype is ambiguous. Similarly, haplotype 6 identifies the introgression at the 3-gene GAL1/7/10 locus, so strains with haplotype 6 at only one of the markers flanking this locus (“chr02_271559” and “chr02_281559”) were also removed. The GAL1/7/10, GAL2, GAL3, GAL4, GAL80 and PGM1 loci were specified using the markers “chr02_271559”, “chr12_292952”, “chr04_463411”, “chr16_80216”, “chr13_171614”, and “chr11_203623”, respectively.

To test the significance of the four-way non-additive interaction linear model between GAL3 and introgressed vs reference alleles of GAL1/7/10, GAL2 and PGM1, GAL3 haplotypes were first discretized into two categories (GAL3+ vs GAL3-). An interaction model involving all four genes was then compared, by ANOVA, to a linear model with an additive effect of GAL3 and interactions between the other three genes.

For estimating the variance explained by markers in the GAL3+ vs GAL3- subpopulations (both lacking the GAL1/7/10 introgression), 100 random training/test strain splits (90:10) were carried out for each marker in each subpopulation. For each marker, an additive model was fit on the training subset of each split. Then the model was applied to the matching test dataset and the residual phenotypic variance was calculated. The mean variance explained and the associated standard error was then calculated across the splits.

For each GAL subpopulation, the effectiveness of global and subpopulation-specific additive models was assessed as follows. For each of the subpopulations (GAL3+, GAL3-, GAL1/7/10 introgression), defined as described above, 100 random training/test strain splits (90:10) were carried out. For each subpopulation and split, an additive model was fit to the training set as described above along with a null (mean only) model. The models were then applied to the matching test datasets and the residuals were calculated. For each of the 100 splits, a global model was also fit after first combining the training strains from each of the three subpopulations. This model was then applied to each test subpopulation individually, and the residuals were calculated.

## CAD statistical and subpopulation analyses

For the major CAD peak on chromosome II, the QTL is close to the right telomere which has an introgression in founder 2. Because of sequence divergence between the introgression and the S288c reference genome, read pairs from strains harboring the introgression (haplotype 2) frequently fail to align to the reference genome, causing undercounting of haplotype 2 at variant sites across this region. To more accurately infer the haplotype this region, read-pairs from each strain that did not align to the S288c reference genome were aligned, using the same parameters used in the original alignment to the reference genome, to the sequence of the right end of chromosome II from founder 2 (chromosome sequence assembly by Plasmidsaurus), corresponding to positions 750,001+ on the reference sequence (732,712+ in the founder 2 chromosome assembly: File #1 in https://doi.org/10.5281/zenodo.17381713). The positions of variant sites from the original sequencing were mapped onto the introgression reference and counts of minor and major alleles were updated using the newly aligned reads as described for the S288c reference sequence, above. Haplotyping of chromosome II was then repeated using the previously described haplotype inference methods. The old and new chromosome II haplotypes were then merged for each strain, replacing the old haplotypes from the right end of chromosome II to the first variant position greater than 750,001 where the original and new datasets call the same parental haplotype. Relative to the original haplotype, some markers are lost from the extreme right telomeric region of chromosome II, as this region is not present in the introgressed sequence. Strains were then split into PCA1 subpopulations based on their haplotype at scaffolding marker “chr02_794058”.

For both the GAL and CAD subpopulation analyses, QTL mapping was carried out as described. For each mapped (sub)population, a 1% significance threshold was calculated from a null distribution generated from 1000 permutations of the data. In each permutation, the haplotype-phenotype assignments were randomized and the maximum LOD score was calculated for the (single) phenotype of interest. Additive models were fit as described.
